# Integration of Hi-C with short and long-read genome sequencing reveals the structure of germline rearranged genomes

**DOI:** 10.1038/s41467-022-34053-7

**Published:** 2022-10-29

**Authors:** Robert Schöpflin, Uirá Souto Melo, Hossein Moeinzadeh, David Heller, Verena Laupert, Jakob Hertzberg, Manuel Holtgrewe, Nico Alavi, Marius-Konstantin Klever, Julius Jungnitsch, Emel Comak, Seval Türkmen, Denise Horn, Yannis Duffourd, Laurence Faivre, Patrick Callier, Damien Sanlaville, Orsetta Zuffardi, Romano Tenconi, Nehir Edibe Kurtas, Sabrina Giglio, Bettina Prager, Anna Latos-Bielenska, Ida Vogel, Merete Bugge, Niels Tommerup, Malte Spielmann, Antonio Vitobello, Vera M. Kalscheuer, Martin Vingron, Stefan Mundlos

**Affiliations:** 1 Max Planck Institute for Molecular Genetics, RG Development & Disease, Berlin, Germany; 2 Institute for Medical and Human Genetics, Charité Universitätsmedizin Berlin, Berlin, Germany; 3 Max Planck Institute for Molecular Genetics, Department of Computational Molecular Biology, Berlin, Germany; 4 CUBI—Core Unit Bioinformatics, Berlin Institute of Health, Berlin, Germany; 5 Charité—University Medicine Berlin, Berlin, Germany; 6 Laboratoire national de santé, Dudelange, Luxembourg; 7 UFR Des Sciences de Santé, INSERM-Université de Bourgogne UMR1231 GAD « Génétique des Anomalies du Développement », FHU-TRANSLAD, Dijon, France; 8 Unité Fonctionnelle d’Innovation diagnostique des maladies rares, FHU-TRANSLAD, CHU Dijon Bourgogne, Dijon, France; 9 Department of Genetics and Centres of Reference for rare disorders, developmental abnormalities and intellectual disabilities, FHU TRANSLAD and GIMI Institute, University Hospital Dijon, Dijon, France; 10 Department of Medical Genetics, University Hospital of Lyon, 69007 Lyon, France; 11 Medical Genetics, Department of Molecular Medicine, University of Pavia, Pavia, Italy; 12 Genetica Clinica, Dipartimento di Pediatria, Università di Padova, Padova, Italy; 13 Medical Genetics Unit, Meyer Children’s University Hospital, Florence, Italy; 14 Medical Genetics Unit, University of Cagliari, Cagliari, Italy; 15 Praxis für Humangenetik, Kinderzentrum Dresden-Friedrichstadt, Dresden, Germany; 16 Department of Medical Genetics, University of Medical Sciences in Poznan, Poznan, Poland; 17 Department for Clinical Medicine, Aarhus University, Aarhus, Denmark; 18 Wilhelm Johannsen Center for Functional Genome Research, Department of Cellular and Molecular Medicine, University of Copenhagen, Copenhagen, Denmark; 19 Institute of Human Genetics, University Hospitals Schleswig-Holstein, University of Lübeck and Kiel University, 23562 Lübeck, 24105 Kiel, Germany; 20 DZHK (German Centre for Cardiovascular Research), partner site Hamburg/Lübeck/Kiel, 23562 Lübeck, Germany

**Keywords:** Gene regulation, Computational biology and bioinformatics, Structural variation

## Abstract

Structural variants are a common cause of disease and contribute to a large extent to inter-individual variability, but their detection and interpretation remain a challenge. Here, we investigate 11 individuals with complex genomic rearrangements including germline chromothripsis by combining short- and long-read genome sequencing (GS) with Hi-C. Large-scale genomic rearrangements are identified in Hi-C interaction maps, allowing for an independent assessment of breakpoint calls derived from the GS methods, resulting in >300 genomic junctions. Based on a comprehensive breakpoint detection and Hi-C, we achieve a reconstruction of whole rearranged chromosomes. Integrating information on the three-dimensional organization of chromatin, we observe that breakpoints occur more frequently than expected in lamina-associated domains (LADs) and that a majority reshuffle topologically associating domains (TADs). By applying phased RNA-seq, we observe an enrichment of genes showing allelic imbalanced expression (AIG) within 100 kb around the breakpoints. Interestingly, the AIGs hit by a breakpoint (19/22) display both up- and downregulation, thereby suggesting different mechanisms at play, such as gene disruption and rearrangements of regulatory information. However, the majority of interpretable genes located 200 kb around a breakpoint do not show significant expression changes. Thus, there is an overall robustness in the genome towards large-scale chromosome rearrangements.

## Abstract (web summary)

Here the authors characterize structural variations (SVs) in a cohort of individuals with complex genomic rearrangements, identifying breakpoints by employing short- and long-read genome sequencing and investigate their impact on gene expression and the three-dimensional chromatin architecture. They find breakpoints are enriched in inactive regions and can result in chromatin domain fusions.

## Introduction

Genomic rearrangements, also called structural variants (SVs), contribute to a large extent to genomic variability and are a common cause of genetic disease. Despite advances in genome sequencing (GS) technologies, their detection remains a challenge, particularly in complex cases with many nested rearrangements. Furthermore, long-read and short-read SV pipelines show different sensitivity and specificity, depending on the type and the size of the SV^1,2^. The interpretation of SVs with respect to pathogenicity has been the subject of many studies, and multiple algorithmic attempts have been made to account for the many effects SVs can have. While changes in copy number may exert their effects via a change in gene dosage, others disrupt genes or result in gene fusions. Furthermore, large-scale rearrangements can alter the three-dimensional chromatin architecture, e.g., by disrupting or reshuffling topologically associating domains (TADs), thereby rewiring gene regulatory landscapes^3^. Even though this mechanism has been observed in congenital malformation disorders^4,5^, as well as for cancer^6^, it is unclear how generalizable the effect of TAD disruptions on gene expression is and how they might be related to an individuals’ phenotype. Conversely, genome-wide depletion of cohesin and CTCF, both important for the formation of TAD boundaries, did not lead to large-scale changes in gene expression^7,8^, and the disruption of a TAD boundary alone does not necessarily lead to a change in gene expression^9^. Furthermore, only a small fraction of genes become misregulated upon disruption of TADs in shattered balancer chromosomes in *Drosophila melanogaster*^10^.

**Fig. 1:**
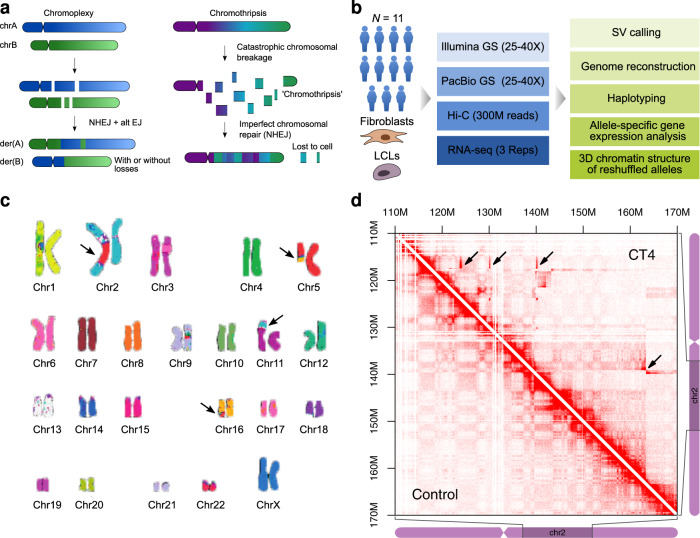
Complex genomic rearrangements investigated in this study. **a** Forms of complex genomic rearrangements: Chromoplexy is characterized by the exchange of larger fragments between chromosomes. Chromothripsis is characterized by a shattering of one or several chromosomal fragments followed by an imperfect repair. Schematic is based on^11,57^. **b** Outline of the study: cohort, clinical sample available, sequencing technologies, and analyses. Schematics of cells were created with BioRender.com. **c** Multicolor FISH of sample CT4 indicating several translocations between chr2, chr5, chr11, and chr16. **d** Hi-C map of chr2 of CT4 showing several large-scale rearrangements. Ectopic interactions are only visible in the CT4 sample (examples indicated by arrows, upper triangular matrix), but not the in a control (lower triangular matrix).

We approached these open questions by studying extreme cases of chromosomal rearrangements, as they are observed in congenital chromoanagenesis. Chromoanagenesis is an umbrella term for complex large-scale genomic rearrangements, which can further be divided into chromoplexy (Fig. 1a), chromothripsis (Fig. 1a), and chromoanasynthesis, depending on the complexity of the events and the gain/loss of genetic material^11^. Complex genomic rearrangements (CGR) are frequent in many cancer genomes^12,13^ but are rare in congenital disorders^11,14^. Despite the massive rearrangements, some affected individuals show only mild clinical symptoms. The complex nature of these rearrangements with many breakpoints and their highly nested form makes it exceedingly difficult to resolve them. They can thus be considered a perfect test bed for technologies to detect and interpret genomic rearrangements.

Here, we investigate the genomes of 11 individuals with complex constitutional chromosome rearrangements, including chromothripsis. We perform an extensive characterization of breakpoints using a combination of short-read (Illumina) and long-read (PacBio CLR) genome sequencing, as well as Hi-C (Fig. 1b). We use these three technologies jointly to detect breakpoints, remove likely false-positive calls, reconstruct shattered chromosomes, characterize the 3D chromatin landscape and investigate novel adjacencies created upon the genomic rearrangements. The analysis of single nucleotide polymorphisms (SNPs) in conjunction with PacBio and Hi-C reads *is* further used for an allele-specific quantification of RNA-seq data from patient cells. The results indicate that the fraction of genes with altered expression is associated with the distance of the gene to a breakpoint.

## Results

### Combining genome sequencing and Hi-C reveals the complexity of genomic rearrangements in germline chromoanagenesis

Ten out of eleven individuals included in this study presented with intellectual disability (ID), while one did not present any pathogenic feature. Their diagnostic workup included karyotyping and microarray-based comparative genomic hybridization (array CGH) (Supplementary Table 1). One case was tested with multicolor FISH, confirming the presence of several translocations (Fig. 1c). Lymphoblastoid cell lines (LCLs) were available for 10 cases, and fibroblasts for one case. To further characterize the rearrangements in detail, we applied Illumina short-read as well as PacBio long-read GS (CLR) (Fig. 1b).

**Fig. 2:**
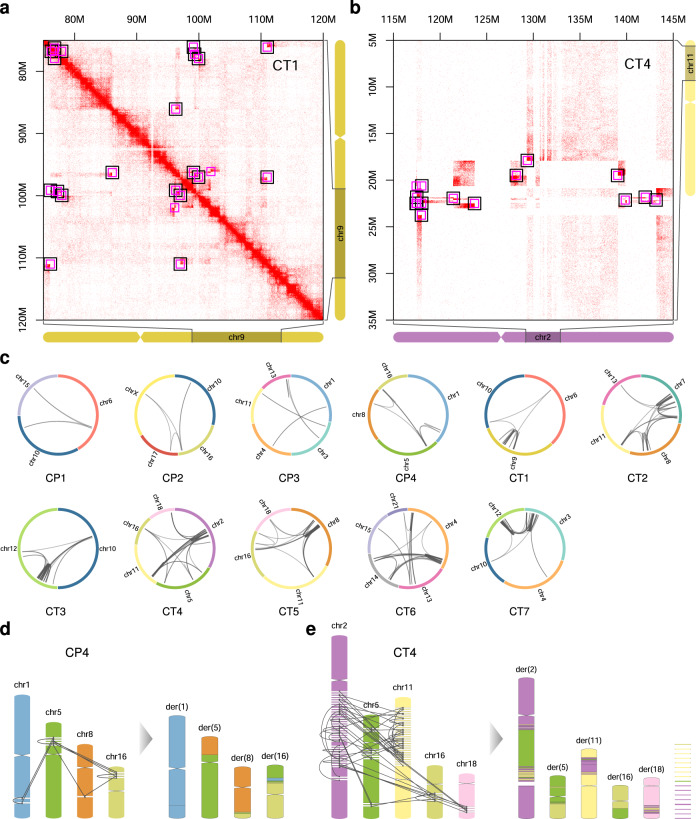
Reconstruction of shattered chromosomes. **a** Overlay of curated SV calls from Illumina GS (pink squares) and PacBio GS (black squares) for a cis Hi-C map as well as for **b** a trans Hi-C map. **c** Circos plots for 11 samples showing all curated large-scale novel adjacencies. **d** Reconstruction graph and derivative chromosomes for CP4. The chromosome reconstruction strategy comprises several steps starting with (i) the placement of all curated breakpoints to generate chromosomal fragments, (ii) connecting chromosomal fragments according to SV calls, (iii) tracing all possible paths in the fragment graph to obtain derivative chromosomes. **e** Reconstruction graph and derivative chromosomes for CT4. The last column shows leftover singletons. Note, small fragments and telomeric ends are shown enlarged.

We first applied the structural variant caller SVIM^15^ to PacBio long-read GS data and detected, on average 243 large-scale novel adjacencies (i.e., >100 kb between the fused positions or trans) per sample when filtering for a quality-value of at least 5 (i.e., 5% of the mean alignment coverage). Large-scale rearrangements create novel adjacencies that cause ectopic interaction patterns in Hi-C maps^16,17^ (Fig. 1d). A novel adjacency becomes especially prominent in the Hi-C map when the genomic distance between the fused positions is large and when the fused chromosomal fragments are large. True novel adjacencies between large fragments colocalize with an ectopic chromatin interaction pattern in the Hi-C map, whereas those that show no evidence of ectopic interaction are likely false positives. We, therefore, focused on novel adjacencies of >100 kb between the fused positions and projected them onto the Hi-C maps (Fig. 2a, b).

After the manual curation of the initial SV calls (See Methods), we revised our SV filtering strategy for PacBio to reduce the call set further and excluded SV calls with at least one of the criteria described in the Methods section. Additionally, we projected Illumina GS large-scale SV calls (>100 kb or trans) from the Delly tool^18^ onto the Hi-C map and curated the SV calls from PacBio and Illumina jointly. Besides the coordinates, the strand information of the novel adjacency can also be informative, because it indicates the direction (Supplementary Fig. 1a), in which the ectopic Hi-C signal is expected^19^. The joint curation of PacBio and Illumina-based calls yielded between 4 and 73 large-scale novel adjacencies per case, most of them detected by both methods (Supplementary Fig. 1b). Only a small subset of calls was unique to one of the methods. We did not consider small-scale novel adjacencies (<100 kb) or novel adjacencies not related to complex rearrangements. However, small-scale novel adjacencies (1–100 kb between anchor points) were considered later in the reconstruction of derivative chromosomes, when their coordinates matched the anchor points of curated large-scale novel adjacencies (See Methods).

### Genome sequencing analysis reveals two regimes of genomic complexity

The analysis of novel adjacencies revealed large differences between the investigated cases in the number of breakpoints, as well as in their distribution throughout the genome. Four cases showed patterns of chromoplexy (hereafter named CP1–CP4), and seven cases exhibited chromothripsis-like structures (CT1–CT7) (Fig. 2c). The chromoplexy cases were characterized by a lower number of large-scale novel adjacencies (4–11) (Fig. 2c) and less complex ectopic Hi-C patterns (Supplementary Fig. 2c, e). Often two or more chromosomes were involved in the rearrangement, with no or only a few cis junctions (Supplementary Fig. 2e). The resulting chromosomal fragments were usually large (Supplementary Fig. 3A), and no copy number gain of fragments was observed. One case (CP3) showed a loss of 6 Mb (Supplementary Fig. 3b). The chromothripsis cases, in contrast, had a higher number of large-scale novel adjacencies (16–73) (Fig. 2c, Supplementary Fig. 1b) and the resulting chromosomal fragments were often smaller (Supplementary Fig. 3a), due to a clustering of breakpoints, resulting in regions of shattering (Fig. 2c). The rearrangements occurred in nested conformation, leading to complex contact patterns in the corresponding Hi-C maps (Supplementary Fig. 2d, f). In some cases, the exchange of genetic material between the chromothriptic patches of several chromosomes was observed as an ectopic signal in the trans Hi-C maps (Supplementary Fig. 2d). The number of affected chromosomes ranged between two and five. Losses of chromosomal fragments were frequent (between 1 and 46 deleted fragments) (Supplementary Fig. 3b), and copy number gains of small fragments (<1 kb) were observed in two cases (Supplementary Fig. 3b). We compared the curated SVs to the database gnomAD-SV^20^ and did not find SVs with matching coordinates (tolerance of the breakpoints ±1 kb and allele frequency >0.01).

To identify SV-breakpoints that are potentially disease-causing, we searched for genes associated with intellectual disability (ID), the most prevalent phenotype in our cohort, that was hit by a curated small- or large-scale SV (Supplementary Table 2). Six out of eleven cases harbor breakpoints within genes associated with ID (Supplementary Table 2). For instance, we detected a breakpoint in GRIN2B (CT3), which was previously identified in this case and described as causative^21^. Moreover, further breakpoint*s* affecting SOX5 were identified, which might contribute to the phenotypic spectrum in this case. Furthermore, we identified a breakpoint in USP7, which is likely to explain the CP4 phenotype. All other genes associated with ID in our cohort were inherited in an autosomal recessive fashion and were therefore discarded as candidates.

### Novel genomic adjacencies are enriched in chromatin at the nuclear periphery

**Fig. 3:**
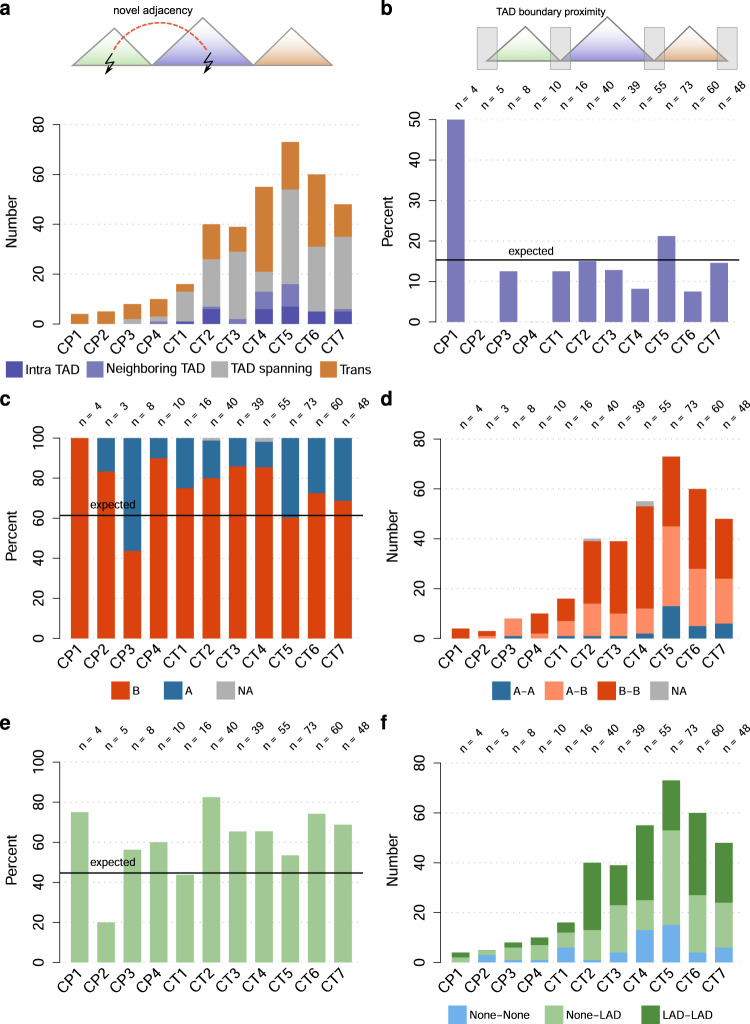
Analysis of the 3D chromatin structure around breakpoints. For selected genomic features, a horizontal line indicates the expected value, which is derived from the genome-wide fraction of the corresponding feature. **a** Number of novel adjacencies with breakpoints located in the same TAD, in neighboring TADs, spanning at least one TAD and on different chromosomes, respectively. **b** Percentage of breakpoints locating in TAD boundary regions (±50 kb). The black line indicates the percentage expected by chance. N is the number of novel adjacencies evaluated. **c** Fraction of compartment type of the breakpoint location for individual samples. The expected line shows here the genomic fraction of the B-compartment. **d** Number and type of compartment fusions induced by the large-scale novel adjacencies. **e** Fraction of breakpoints located in LADs. The black line indicates the percentage expected by chance. **f** Number and type of LAD/Non-LAD fusions.

We investigated the occurrence of novel adjacencies with respect to known features of the genome and chromatin organization, such as TADs, A/B compartments, and lamina-associated domains (LADs)^22^. To evaluate the enrichment of breakpoints with respect to genomic features, we computed *P*-values based on empirical background models, such as novel adjacencies with random coordinates (See methods and Supplementary Fig. 18). Novel adjacencies are potentially TAD disrupting, as they may fuse the regulatory content of different TADs (Fig. 3a), potentially causing gene misregulation^3^. According to this, evolutionary rearrangement breakpoints of different vertebrate species were found to be enriched at TAD boundaries. Breaks within TADS are thus being avoided by negative selection^23^. We thus tested if TAD boundaries are enriched for breakpoints (Fig. 3b), but did not observe an enrichment (*P*-value = 0.819).

An important layer of chromatin organization is the segregation of chromatin into A and B compartments. Compartments are defined based on Hi-C maps^24,25^, showing preferential long-range interactions between regions of the same compartment type. A and B compartments were observed to largely overlap with the transcriptional state and epigenetic features of euchromatin and heterochromatin, respectively. Interestingly, we observed an enrichment of breakpoints in the B-compartment (*P*-value = 0.037) (Fig. 3c). Besides the fusion of two B-compartment locations, we also observed the fusion of an A-compartment locus to a B-compartment locus (Fig. 3d). In line with this observation, we investigated the abundance of breakpoints in lamina-associated domains (LADs). LADs are chromatin domains interacting with the nuclear envelop and can be detected by DamID-seq^26,27^. They are generally transcriptionally less active than non-LAD regions of the genome. Similar to the A/B-compartment analysis, we observed an enrichment of breakpoints in LADs (*P*-value = 0.008) (Fig. 3e). LAD-LAD fusions are an abundant type of novel adjacencies (Fig. 3f). Additionally, also a fusion between regions that are LAD and non-LAD regions in a WT genome occurred (Fig. 3f).

An analysis of repeats at breakpoints did not show a general preference of breakpoints to occur in repeats (*P*-value = 0.879) (Supplementary Fig. 4a). Cases CP2, CP4, and CT1 show values above the expected value, but have relatively few breakpoints in general. An analysis of repeat classes overlapping with breakpoints did not reveal a striking, recurring pattern that would be shared by several cases (Supplementary Fig. 4b).

An in-depth analysis of the flanking sequences around the breakpoints (See Methods) showed that novel adjacencies are often associated with small losses or gains of genetic material indicating imperfect fusion of chromosomal fragments (Supplementary Fig. 5). Additionally, we searched for microhomology, i.e., short regions of DNA sequence homology^28^, around the breakpoints of novel adjacencies. The analysis revealed only low degrees of microhomology (Supplementary Fig. 5b), indicating that sequence homology is a minor aspect of the fusion events.

In summary, we observed an enrichment of breakpoints in the B-compartment and LADs (regions with low gene expression), while we found no striking association with TAD boundaries and repeat regions.

### Reconstruction of whole derivative chromosomes

Next, we used the high-confidence set of novel-adjacency calls to build a reference-based reconstruction graph of the derivative chromosomes. In the first step, we used the curated breakpoint positions to split the original WT chromosomes into the corresponding chromosomal fragments (Fig. 2d). In a reconstruction graph, each chromosomal fragment defines two nodes, i.e., at the 5′ and at the 3′ end of each fragment. The 5′ and 3′ nodes of the same fragment are implicitly connected by an edge. Fragments with a telomere have only one node on the non-telomeric side. All high-confidence novel adjacencies were included as additional edges to the reconstruction graph. Afterward, the reconstruction graph was traversed, and the order and orientation of chromosomal fragments along the paths revealed the layout of the derivative chromosome.

In chromoplexy cases, which are depleted of cis novel adjacencies, the reconstruction yielded whole derivative chromosomes (Fig. 2d). For most of the complex chromothripsis cases (CT2, CT4–CT7), the reconstruction graph was not complete because not all novel adjacencies could be identified leading to missing junctions. In these cases, the reconstruction stops at the stage of incomplete chromosomal scaffolds (Fig. 2e). To overcome this problem, we included Hi-C information to complement the reconstruction analysis. In the Hi-C derived interaction matrix, we used a 2D grid defined by the breakpoints and searched within this grid for ectopic Hi-C contacts shared between scaffolds. In case of ectopic interactions, the corresponding scaffolds can be further grouped together, assuming their origin from the same derivative chromosome (See Methods, Supplementary Fig. 6). The principle also works in reverse; for checking reconstructions, the pattern of the derivative chromosome can be projected back onto the Hi-C map to determine if it is compatible with the ectopic Hi-C patterns. The grid representation of breakpoints has another advantage because it indicates that breakpoints are missing when sharp edges of ectopic Hi-C patterns are not bordered by a breakpoint line. These indicators provide valuable information about derivate chromosome reconstruction.

After grouping incomplete scaffolds to putative derivative chromosomes (Supplementary Fig. 6), Hi-C was instrumental to identify the order and orientation of the components. For the majority of cases, the derivate chromosomes could be readily resolved with only a few large components remaining (Supplementary Fig. 7). For a small number of components, all possible solutions can be enumerated in a permutation approach (See Methods, Supplementary Fig. 8) and each of the solutions can either be inspected visually for its plausibility, or it can be evaluated with a scoring function to identify the best solution.

The reconstruction graph also contains leftover fragments which have no connecting edge (Fig. 2e, Supplementary Fig. 7). These singletons are candidates for a loss of genetic material. When checking the coverage of Illumina GS, indeed, many of these fragments appear as deletions (Supplementary Fig. 2, Supplementary Fig. 10). The remaining fragments, usually very small (<5 kb), seem to be still present in the genome, but their location remained unknown. Rarely, fragments of the reconstructed derivative chromosomes have low coverage, suggesting that they are rather deleted than part of the derivative chromosome (Supplementary Fig. 10). For case CT7, it was not possible to place the telomeric part of the q-arm of chr12 based on Hi-C, even though the fragment does not appear to be deleted.

### Chromosomal rearrangements are associated with changes in gene expression based on genomic distance

**Fig. 4:**
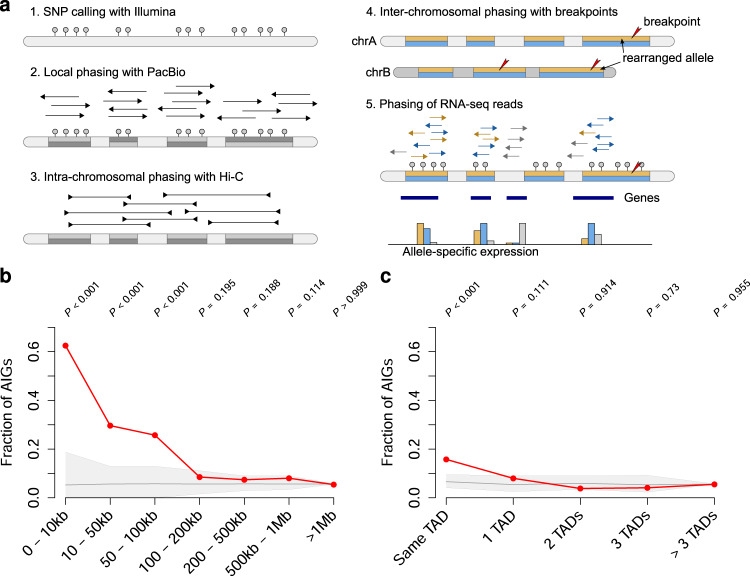
Haplotyping and allele-specific analysis of RNA-seq data. **a** Haplotyping: Based on SNPs from Illumina sequencing larger haplotype blocks are created using PacBio long-reads. Large haplotype blocks are connected with the help of Hi-C. The breakpoints of rearrangements are also phased and used to label WT allele and affected allele. The haplotype information is used to phase RNA-seq data at positions with informative SNPs and to derive allele-specific gene expression values. **b** Differential gene expression analysis around breakpoints: Fraction of allelic imbalance genes (AIGs) with respect to the distance between an expressed gene and the closest breakpoint (red line). As a control, simulations of random breakpoints were performed. The light gray area with the gray line indicates the 5th percentile, median and 95th percentile, respectively, expected by chance. *P*-values were computed by comparing each observed value against values obtained from an empirical background model. The tests were performed right-sided, adjustments for multiple testing were not performed. **c** Same as **b**, but the distance of a gene to the next breakpoint is measured in TAD units (Same TAD, 1 TAD, etc.). *P*-values were computed as described in **b**.

To quantify the effect of chromosomal rearrangements on gene expression, we haplotyped the genomes using single nucleotide polymorphisms (SNPs) from Illumina GS, SV calls from PacBio together with PacBio long-reads and Hi-C reads, in order to produce allele-specific expression data (Fig. 4a). After phasing the RNA-seq data, DESeq2^29^ was used to compare the expression of the rearranged allele vs. the WT allele (See Methods). Thus, for each individual, the expression was analyzed within the sample, instead of compared across individuals. Only genes which are expressed on at least one allele and for which the phasing of RNA-seq data was possible are informative (Supplementary Fig. 9b). Overall, we found 70 genes with the transcription start site (TSS) within 100 kb to the nearest breakpoint and phased RNA-seq signal. Out of these, 22 genes showed allelic imbalance expression (abs(Log2FC) > 1 and padj < 0.05). Simulations with random breakpoints showed that the effect is statistically significant up to a distance of 100 kb from the breakpoint and then decreases quickly (Fig. 4b). As an alternative approach, we performed simulations with permuted gene expression tables instead of random breakpoints and observed similar statistical trends (Supplementary Fig. 20). For distances up to 1 Mb, the fraction of allelic imbalanced gene expression appeared elevated, but was not statistically significant. In short, we observed an association between AIG and breakpoint proximity, and we further investigated the cause of this pattern.

Gene disruption is a likely explanation for reduced gene expression (Supplementary Fig. 9a, Supplementary Fig. 11a), and indeed, we found 39 informative genes with an intragenic breakpoint. However, not all of them showed downregulation: We also observed unchanged expression or even upregulation (Supplementary Fig. 11a). For genes hit by a breakpoint, we observed, that not necessarily all transcript variants were truncated by the breakpoint (Supplementary Fig. 12). Upregulation of gene expression could be caused by TAD fusions and/or enhancer–promoter rewiring. Alternatively, fusion transcripts, for which we found evidence on RNA-seq level (Supplementary Data 1), could also result in upregulation. These features can be overlapping; e.g., for seven genes, we found allelic imbalanced expression, an intragenic breakpoint, and a predicted fusion transcript at the same time (Supplementary Fig. 13).

In the group of upregulated genes, the fraction of housekeeping genes appears to be lower than expected by the genome-wide fraction of housekeeping genes (Supplementary Fig. 11b).

When grouping genes not by genomic distance to the closest breakpoint but by measuring the distance in the number of separating TADs, the enrichment of allelic imbalance genes (AIGs) was less pronounced (Fig. 4c), supporting the concept that genomic distance has a stronger effect on gene expression alterations than separating TADs.

Lastly, the majority of the breakpoints (69%) did not have an informative gene with its TSS in the region ±200 kb around the breakpoint (Supplementary Fig. 14). However, out of the 31% of breakpoints with at least one informative gene in ±200 kb proximity, 45% have at least one AIG (Supplementary Fig. 14).

### Reshuffled Hi-C maps of derivative chromosomes reveal abundant TAD fusion events

**Fig. 5:**
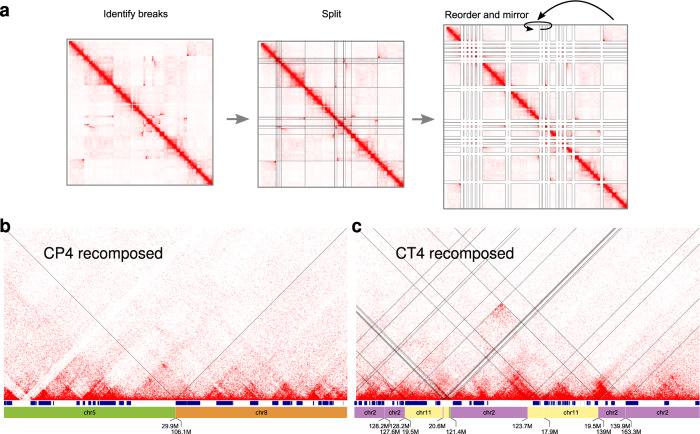
Analysis of the chromatin structure of reshuffled chromosomes. **a** Recomposing of Hi-C maps by cutting a Hi-C map at all breakpoint positions and reordering and reorienting all rows and columns according to the reconstruction scheme. **b** Recomposed Hi-C map for CP4 (**c**) and CT4. Labels for small chromosomal fragments are not shown to improve clarity. Note, the subtraction of a Hi-C map from a control sample could not remove Hi-C patterns of the WT allele entirely, visible in the map for CT4.

Next, we sought to evaluate the rearranged 3D chromatin structure pattern in *chromoanagenesis*. The reconstruction of shattered chromosomes provides the linear layout of the derivative chromosomes, i.e., the location of functional elements such as genes, known regulatory elements, and insulators. The reconstructed derivative chromosomes can also be used to rearrange the Hi-C map accordingly (Fig. 5a). This recomposition brings the Hi-C signal from rearranged chromosomes into order, resolving the Hi-C patterns from rearrangements, which have been visible before. Interestingly, the derivative Hi-C maps reveal chromatin structures that emerged upon the rearrangements, such as TAD fusions and loops (Fig. 5b, c). We checked the rearranged Hi-C maps for TAD fusion events, focusing on candidate locations that have sufficient Hi-C signal and are interpretable. 86% of these locations in the chromoplexy cases and 80% in the chromothripsis cases showed also in the Hi-C map evidence for a TAD fusion. Taken together, massive chromosomal rearrangements in germline chromoanagenesis disturb gene expression. However, this occurs only to a certain degree, with most of the observable genes located in close proximity to a breakpoint not changing their expression. Thus, in our cohort, we observed overall robustness of the genome towards constitutional large-scale chromosome rearrangements.

### Evaluation of the accuracy of reconstructed derivative chromosomes

We next evaluated the accuracy of the reconstructed derivative chromosomes using different approaches *because* a comprehensive ground truth is missing. On a coarse scale, karyotyping confirmed the majority of chromosomes we identified as being affected. For CT1 we found no involvement of chrX reported by karyotyping (Supplementary Table 1). Additionally, our approach detected the involvement of chr10 (CP2), chr11 (CP3), chr1 (CP4), and chr18 (CT5) that was not reported by karyotyping.

A previous study on case CT2 using mate-pair sequencing and Sanger sequencing^30^ reported 41 novel adjacencies, the same number we curated. 37 novel adjacencies are common with our set, while 4 are unique to each of the sets when requiring a distance <1 kb between breakpoints and the same strand orientation at the junction to define common novel adjacencies.

As a next evaluation approach, we mapped the PacBio long-read data to custom genomes generated based on our reconstructions. Next, we checked, if PacBio long-reads span breakpoint junctions predicted by our curated novel-adjacency set. For 365/376 junctions, one or several supporting PacBio reads could be found (Supplementary Fig. 19b). For 9/11 cases, all tested junctions are covered by junction-spanning reads (Supplementary Fig. 19b). For case CT2, a single junction has no supporting PacBio read, but the fraction of junction-spanning reads is, for unknown reasons, very low for this sample in general, which also leads to large deviations in the SV calling when comparing Illumina-based and PacBio-based SV calling (Supplementary Fig. 1b). For the most complex case CT5, 10 junctions had no support by spanning PacBio reads. This indicates that, on a local level, the reconstruction still contains several errors. Reconstruction errors might be attributed to different reasons, such as (i) false-positive novel adjacencies in the curated novel-adjacency set used for the reconstruction, (ii) missed complexity by undetected SVs or not considered SVs, (iii) in rare cases, the breakpoint position indicated by SVIM had an offset to the real breakpoint. This can also lead to deviations in the reconstruction process, especially when the fragments are very small, as in the case of CT5. Additionally, also the analysis of the Illumina GS coverage (Supplementary Fig. 10) indicates missing breakpoints because a few fragments which appear deleted based on coverage are still present in the reconstructed derivative chromosomes.

The last evaluation we performed is the reprocessing of the Hi-C data with custom genomes. This approach is similar to the recomposition of the Hi-C map described above but starts already at the level of the genomic sequence. We did not observe larger misjoins in cis parts of the custom Hi-C maps, which would usually be accompanied by a discontinuity of the Hi-C signal (Supplementary Data 2).

## Discussion

The first step towards a better understanding of the implications of complex rearrangements on chromatin structure, gene regulation, and phenotype is the comprehensive detection of all breakpoints and reconstruction of shattered chromosomes. In previous studies of germline CGRs, breakpoints were detected using mate-pair sequencing, followed by filtering for sample-specific SVs and subsequent validation of breakpoints using PCR and Sanger sequencing^30,31^. Here, we combined Illumina and PacBio GS with Hi-C to identify breakpoints and resolve chromosomal rearrangements in 11 cases with CGRs. The majority of novel adjacencies with large distances between the breakpoints were detected by the Illumina-based caller, as well as the PacBio-based caller.

In general, GS methods suffer from a high false-positive rate when using the initial call set of common tools such as Delly^18^ or SVIM^15^. In contrast to GS, Hi-C probes the 3D interactions of the genome and projects them onto a two-dimensional map^24^. We used this property of Hi-C for independent validation of large-scale SV-breakpoints^16^ detected by Illumina and/or PacBio GS. Additionally, Hi-C is a powerful tool to order and orient scaffolds^32–34^. The disentangling of genomic rearrangements based on Hi-C was suggested for developmental diseases^17^ and demonstrated in an automated manner in cancer cell lines^35^. The use of Hi-C for a manual reconstruction of derivative chromosomes was described recently for somatic chromothripsis induced in a cell line^19^. Here, we combined Hi-C with SV calls from short and long-read sequencing to reconstruct germline shattered chromosomes, which was possible for whole chromosomes in many instances. In the remaining cases, a reconstruction was possible to the level of large chromosomal blocks, which were grouped further to derivative chromosomes. By applying a permutation approach, a chromosome-wide reconstruction was achieved. However, this came with more uncertainty, because no direct evidence for the junction between chromosomal fragments was available, and the details of the junction remain unclear.

The approach presented here has some limitations. We analyzed only large novel adjacencies >100 kb or translocations and added afterward a few smaller novel adjacencies (1–100 kb) to the reconstruction. However, we did not consider the complexity of rearrangements from novel adjacencies with <1 kb between the fused positions. The presence of small rearranged fragments can be overlooked by Hi-C and make an evaluation difficult. For example, the local shattering of CT5 had such a high complexity that the reconstruction approach reached its limits. In some cases, Illumina- and PacBio-derived breakpoint positions disagreed by more than 50 bp. Especially when breakpoints are very close to each other, this can create ambiguity and alter the reconstruction. To match breakpoints in the reconstruction, we allowed a tolerance of <50 bp. With this tolerance, we obtained sometimes more than one junction per fragment end. We resolved these conflicts by removing the novel adjacency, which had the lowest evidence, i.e., was found by one technology only or had poor support in Hi-C. As the judgment of novel adjacencies based on Hi-C is difficult for small fragments, different reconstructions are possible, depending on which novel adjacencies are removed in case of ambiguity. The presence of some remaining reconstruction issues and missed novel adjacencies are indicated by mapping PacBio long-reads to custom genomes (Supplementary Fig. 18 and section ‘Evaluation of the accuracy’), by the reconstructed Hi-C maps, as well as by fragments, which appear deleted based on coverage, but are still in the reconstruction (Supplementary Fig. 10). Additionally, copy number gains can create ambiguity in the reconstruction and imply chromosomal fragments with overlap, which would not be properly handled here. As Hi-C is based on short-read mapping, regions with low mappability, such as centromeres, have poor or missing Hi-C signals hampering the evaluation of rearrangements in these regions. The reconstruction of derivative chromosomes, as well as the assignment of haplotypes for phasing RNA-seq data, is based on the assumption that all large-scale rearrangements occurred in the same allele and were manifested in the germline. A useful extension of our approach could be the testing for polymorphisms. During the recomposition, the different pieces of the Hi-C map are assembled, but no additional normalization for the individual pieces, such as proposed in ref. 35 was implemented. The recomposition of the Hi-C map, as well as the permutation of possible reconstructions, was limited to fragments that cover a complete Hi-C bin (25 and 100 kb, respectively); smaller fragments were removed for these steps. An alternative approach without the issues of small fragments is the mapping of Hi-C reads to a custom rearranged genome as performed for the evaluation of our reconstructions (Supplementary Data 2).

Our curated set of novel adjacencies provided the basis for an in-depth analysis of the rearranged chromosomes, the distribution of the breakpoints, and their effect on gene expression. The breakpoints showed no preference for TAD boundaries, in contrast to what was reported for rearrangements which manifested over the course of evolution^23^. However, we did observe an enrichment of breakpoints for the B-compartment as well as for LADs. These transcriptionally inactive regions of the genome are located closer to the nuclear periphery and may thus be prone to damage from defective isolation of the nucleoplasm or being more tolerant towards rearrangements. It is important to note, that we are looking here only at a small number of samples, limiting the possibility of drawing general conclusions on enrichments of breakpoints with respect to genome organization.

The combination of Illumina GS, PacBio GS, and Hi-C allowed haplotyping of the patients’ genomes. By phasing RNA-seq data, we were able to investigate the difference in RNA-expression profiles between the intact WT alleles and the shattered alleles. The majority of genes did not show a significant difference in the allelic balance, suggesting that many large-scale rearrangements had no effect on the investigated genes and cell type. However, we observed an enrichment of regulated genes within a region of 100 kb around breakpoints. At larger distances up to 1 Mb, the level of AIGs appeared still elevated, although not statistically significant. Most cases of altered gene regulation within 100 kb breakpoint distance were associated with breakpoints within the gene. While gene disruption is a likely scenario for downregulated genes, we observed upregulation for 50% of genes (11/22). For these cases, misregulation by e.g., enhancer adoption is a possible scenario, modulating the expression of intact transcript isoforms, as well as fusion transcripts in some cases as indicated by fusion transcript analysis of RNA-seq data.

The observation that a rather small fraction of genes close to a breakpoint showed allelic imbalanced expression has to be also seen in the context of the experimental setup of this study. The tested lymphoblastoid cell lines represent only a small fraction of potentially regulated genes, and the number of observable genes is further reduced because phasing RNA-seq could only be done at SNP positions. During development and in all tissues/organs, this will be very different, thus dramatically increasing the number of potentially regulated genes. Nevertheless, the results are in line with a study in *Drosophila*^10^ in which only a minority of genes showed deviations in expression in the presence of large-scale rearrangements in shattered balancer chromosomes. However, as in the *Drosophila* study, it has to be considered that the individuals studied here are survivors, i.e., this is a negative selection against more severe effects. In addition, we acknowledge that the cell lines studied do not reflect the complexities of an embryonic environment in which gene regulation happens at a very different level.

## Methods

### Subjects

From our in-house cohort of individuals with chromosomal rearrangements detected by karyotyping, we selected seven cases with >3 chromosomal rearrangements to be enrolled in this study. Through collaborative efforts, we obtained an additional four cases. In total, 11 constitutional CGRs were selected for this study. Informed consent to publish genomic and clinical data were obtained from all patients (or their legal guardian). The study adhered to the Declaration of Helsinki standards, and was approved by the internal Ethics Committee of the Institute for Human Genetics of Charité—Universitätsmedizin Berlin, Berlin, Germany. Peripheral blood lymphocytes were used for establishing lymphoblastoid cell lines by EBV transformation. Molecular cytogenetics investigations were performed before for CP2^36^ and CT3^21^. For CT3, previous serial FISH mapping found one of the breakpoints to disrupt *GRIN2B*^21^. For CT2 a fibroblast cell line was established and characterized by molecular cytogenetic and mate-pair sequencing in previous studies^30,37^.

### Cell culture

LCLs were cultured in RPMI medium with 15% fetal bovine serum (FBS) and 1% pen-strep. Fibroblasts were cultured in DMEM with 10% FBS, 1% L-glutamine, and 1% pen-strep.

### Illumina genome sequencing

Short-read Illumina whole-genome sequencing (GS; 30× coverage) was performed on DNA isolated from the cell lines. Sequencing was performed by Macrogen (South Korea) on Illumina HiSeq X machines with Illumina TruSeq PCR-free chemistry. After quality control (QC), reads were aligned to the GRCh37 reference genome with BWA-MEM (version 0.7.17)^38^ duplicates were masked using SAMBLASTER (version 0.1.24)^39^, and the reads were sorted and converted to BAM files using Samtools (version 1.9)^40^. SVs were detected using Delly (version 0.8.1)^18^. Coordinates of novel adjacencies were derived from the SV calls considering the classes DUP, DEL, INV, and BND. Bcftools (version 1.10.2)^40^ was used for the processing of VCF files.

### PacBio genome sequencing

We cultured 4 × 10^7^ cells (LCLs and fibroblasts) for PacBio CLR GS, and high molecular weight (HMW) DNA (for >30 kb SMRTbell Libraries) was extracted using a smart DNA prep kit (Analytik Jena). Quality control step was performed using the DNF-467 Genomic DNA 50 kb Analysis Kit on a 5200 Fragment Analyzer system (Agilent). Library preparation: briefly, all samples were sonicated using the Megaruptor 3 shearing kit on the Megaruptor 3 instrument (Diagenode; parameters 20 µg HMW-DNA; Speed: 3). Purification step was performed with AMPure PB Beads (Ratio 0,46×). Library preparation QC was performed using the DNF-464 High Sensitivity Large Fragment 50 Kb kit. We used the kit SMRTbell Express Template Prep Kit 2.0 (100-938-900) and performed size selection using BluePippin Size-Selection System (Sage Science). Range selection mode “BPstart” 30,000 bp, “BPEnd” 80,000 bp with library input of 3–5 µg. Library sequencing was performed on Sequel II system (Pacific Biosciences).

The eleven PacBio CLR datasets were generated on a PacBio Sequel II machine. One sequencing run with a single SMRT cell was performed per patient with the exception of CT3 (2 SMRT cells). We aligned all datasets to the GRCh37 human reference genome using pbmm2 (version 1.3.0, parameters: -preset “SUBREAD”, -median-filter), yielding alignment coverages between ~20× and 40× (Supplementary Fig. 15). Median read lengths varied between 8 kb for patient CT3 and 29 kb for patient CP4.

### PacBio novel-adjacency calling and filtering

Novel-adjacency calls were produced with the SV caller SVIM^15^ (version 1.4.1, parameters: -all_bnds -max_sv_size 5,000,000 -segment_gap_tolerance 300 -segment_overlap_tolerance 100 -zmws). In the all_bnds mode, SVIM collects all novel adjacencies indicated by PacBio read alignments considering adjacencies from translocations, deletions, inversions, interspersed, and tandem duplications. Simple insertions were not considered because the insertion of bases does not create any novel adjacencies between reference loci. Novel-adjacency calls from different reads were clustered using a hierarchical clustering approach based on the sum of distances between breakend positions.

To reduce the rate of false positives calls, we removed novel-adjacency calls matching at least one of the following criteria: (i) low number of supporting reads (below 5% of the mean alignment coverage); (ii) artificially high read coverage; (iii) at gaps in the reference genome; (iv) in proximity to segmental duplications; and (v) SV calls occurring in more than one sample.

In case, we detected a novel adjacency by both technologies at the same location in the Hi-C map with matching strand orientations, we selected the Illumina-based call for further downstream analysis when labeled as ‘precise’ by Delly. Otherwise, we used the PacBio-based call.

The PacBio-based novel adjacencies calls <100 kb are without filtering step (v).

#### Score-based filtering

To further reduce the false-positive rate, adjacencies supported by a low number of reads were removed. We applied sample-specific thresholds to accommodate for the large variance in alignment coverage across samples. To retain high sensitivity, a lenient threshold of 5% of the genome-wide average alignment coverage was used for each sample.

#### Coverage-based filtering

We computed the average alignment coverage in nonoverlapping genomic windows of 10 kb. Windows with an average coverage higher than three times the genome-wide average coverage were annotated as high-coverage regions, and novel adjacencies found in such regions were filtered out.

#### Gap-based filtering

To remove spurious novel adjacencies caused by the presence of gaps in the reference sequence, novel adjacencies with a distance of less than 10 kb to a reference gap were filtered out. Gap locations were detected using seqtk (version 1.3, https://github.com/lh3/seqtk, parameters: cutN -n 1000).

#### Duplication-based filtering

Due to their length and similarity, segmental duplication regions can confuse the read alignment algorithm leading to erroneous alignments. To remove unreliable novel-adjacency calls between related segmental duplication regions, novel adjacencies overlapping annotated segmental duplication regions (source: http://humanparalogy.gs.washington.edu/build37/data/GRCh37GenomicSuperDup.tab) were filtered out.

#### Cohort-based filtering

Finally, the remaining novel adjacencies from all patients were merged, and similar adjacencies were clustered using a breakend distance cutoff of 1 kb. Non-unique adjacencies, i.e., those present in more than one sample, were particularly common in the repetitive genomic regions close to the centromeres and telomeres and were enriched in false positives and population polymorphisms. To retain only genomic rearrangements unique to each patient, adjacencies present in more than one sample were filtered out.

It is noted that the filtering steps could potentially also remove true positive SV calls, e.g., genomic variation can also be close to segmental duplications^41^.

### Preparation of Hi-C libraries

Hi-C libraries were processed as described in the previously published in situ protocol and with minor modification using our in-house modified version^17^. Briefly, ~1 million cells were fixed in 2% formaldehyde, lysed, and digested overnight with DpnII enzyme (New England BioLabs, M0202). PCR amplification (4–8 cycles) using the NEBNext Ultra II Q5 Master Mix (New England BioLabs, M0544). PCR purification and size selection were carried out using Agencourt AMPure XP beads (Beckman Coulter, A63881). Libraries were deep sequenced (~360 million fragments) in 75 bp, or 100 bp paired-end runs on a NovaSeq6000 (Illumina). For each individual, the Hi-C library was created by pooling between two and four technical replicates generated from two different cell cultures, to ensure higher complexity of the sequencing library.

### Hi-C bioinformatics analysis

Paired-end sequencing data were processed using the Juicer pipeline v1.5.6, CPU version^42^ with BWA*-MEM* (version 0.7.17)^38^ for aligning short reads to reference genome hg19. Alternative haplotypes were removed from hg19, and the sequence of the Epstein-Barr virus (NC_007605.1) was added. Replicates were merged by combining filtered and deduplicated read-pairs output from the Juicer pipeline. Juicer tools (version *1.7.5*)^42^ was used to create hic-files for visualization and downstream analysis. Juicebox (Desktop version 1.8.8)^43^ was used for the inspection of raw count maps, as well as maps normalized with Knight and Ruiz (KR) normalization^25,42,44^ at different bin sizes. For the generation of Hi-C maps, we used read-pairs with mapping quality (MAPQ) ≥ 30. However, spotting genomic rearrangements, it can be helpful additions to generate and inspect Hi-C maps with lower, more permissive MAPQ thresholds.

For the display of Hi-C maps in figures, simple raw count maps were used to show ectopic Hi-C patterns. For visualization as heatmaps with a linear scale, high values were truncated to improve visualization. Novel-adjacency calls were overlayed with the Hi-C map as 2D annotation in Juicebox for visual inspection.

### RNA-seq library preparation

RNA extraction was performed in all 11 samples using the RNeasy mini kit (Qiagen, Hilden, Germany). Poly(A) mRNA capture was performed using the KAPA mRNA HyperPrep Kit (KR1352—v5.17), and the RNA-seq was performed on a HiSeq4000 (Illumina) in three technical replicates (PE75, 50 million fragments per sample), except for CT5 for which two technical replicates were available.

### DESeq2 analysis

Raw reads were mapped to the human genome build hs37d5 using STAR (version 020201)^45^ and further filtered for a minimum mapping quality of MAPQ = 5.

Alignments were then further used to distinguish the wild-type (WT) allele and the rearranged allele (See Methods: Halpotyping and Phasing). This resulted in a table with read counts per gene and per allele for each replicate. DESeq2 (version 1.26.0)^29^ was then applied for an adapted differential gene expression analysis by contrasting the read counts from the rearranged allele against the read counts from the WT allele taking all three replicates into account. We used an adjusted *P*-value < 0.05 and an abs(Log2FC) >1 to define allelic imbalance genes.

Sample CT2 was excluded from the analysis of the allelic imbalance genes due to a high base level of allelic imbalance genes (Supplementary Fig. 9C). Additionally, genes on the sex chromosomes were excluded from the analysis of the allelic imbalance. After the DESeq2 analysis, genes for which no padj-value could be computed were not considered further in the analyses.

For computing, the distance between a gene and a breakpoint, the distance between the TSS and the closest curated breakpoint was computed. In case a gene had alternative TSS, the most 5′ TSS was used for the distance computation.

### Fusion transcript detection

We used the software Arriba (version 2.1.0)^46^ with default parameters for the detection of fusion transcripts in RNA-seq. Each RNA-seq replicate was analyzed individually. Afterward, we projected the coordinates of the predicted breakpoints onto the Hi-C map and manually selected fusion transcript candidates, which were located at ectopic Hi-C interaction patterns associated with novel adjacencies or close by. For the downstream analysis, we kept only candidate genes, which were labeled as ‘high confidence’ by the Arriba tool in at least one replicate.

### Haplotyping and phasing

#### Preparation of Hi-C reads for haplotyping

In order to use Hi-C reads for haplotyping, we separately mapped the Hi-C reads of different replicates to the human reference genome hg19. For this, we followed the practice suggested by HapCUT2^47^. We used BWA-MEM (version 0.7.17)^38^ and Samtools (version 1.10))^40^ for mapping single reads of Hi-C pairs and for sorting the aligned files, respectively. We used the Hi-C_repair tool developed in HapCUT2^47^ and Samtools to combine single-read alignment files. We finally used the Picard tools (version 2.20.8-0) (https://broadinstitute.github.io/picard/) for marking duplicates. Finally, we merged the alignment files of different technical replicates into one Hi-C BAM file for each sample.

#### Haplotyping with small variants

PacBio, Illumina, and Hi-C data were integrated to compute the haplotypes. PacBio and Illumina reads were mapped to *the* GRCh37 reference genome (See sections:’PacBio genome sequencing‘ and ’Illumina genome sequencing‘, respectively). We used Illumina reads for variant calling. We parallelized the jobs over the chromosomes to speed up the processes. The following steps were performed on individual chromosomes. First, we called small variants (variants < 50 bp) using freebayes (version 1.2.0)^48^. At this step, we filtered out homozygous variants and those heterozygous variants with *a* calling quality of <30. We then extracted haplotype informative reads, the reads with at least two variants, from PacBio and Hi-C data using extractHAIRS module of HapCUT2. Afterward, Hi-C and PacBio informative reads were merged and fed to HapCUT2 for haplotyping. The HapCUT2 results were converted to the VCF format using Whatshap (version 0.18)^49^. In the next step, *we* tagged the PacBio reads with haplotypes and haplotype blocks using Whatshap. We then merged the variants and tagged PacBio reads of each sample into one phased VCF and one BAM file, respectively. After these steps, the variants are grouped into so-called phase sets.

#### Investigating the feasibility of integrating breakpoints into haplotyping

We clustered long-reads on each breakpoint into three groups, *namely* haplotype one (H1), haplotype two (H2), and unassigned reads (NoHapInfo). For this, we used PacBio reads haplotag, i.e., the reads tagged by the haplotypes. Supplementary Fig. 16 shows a screenshot of the genomics viewer IGV^50^ at a breakpoint (sample: CT4, locus: chr2:140, 217, 329–140, 217, 537). The reads in H1, H2, and NoHapInfo groups are shown in brown, purple, and gray color, respectively. In addition, we analyzed the breakpoints using split-mapped PacBio reads. We clustered the reads into rearranged (in short CGR), wild-type (in short WT), and undecided (NoBpInfo) groups (see Supplementary Fig. 16). We assigned a read into a WT group if the mapping quality ≥20 and the read spans ±90 bp of a breakpoint (dashed red lines in the example shown in Supplementary Fig. 16). We assign a read into a CGR group if *the* mapping quality ≥20 and the read-only pass one side of a breakpoint. In addition to that, a CGR read needs to be mapped to another region in the genome as supplementary alignment. We excluded the reads ending ±90 bp of breakpoints and assigned them to the NoBpInfo group.

There are nine possibilities for tagging the reads on the breakpoints of a phase set. Note that the phase sets may span several breakpoints. Each read can be tagged with H1 or H2, or NoHapInfo. Also, each read can also be assigned to CGR, WT, or NoBpInfo classes. Among the nine groups obtained by combining the two tags, the four groups of WT/H1, WT/H2, CGR/H1, and CGR/H2 are informative for the investigation of the feasibility of haplotyping using breakpoints. We observed that most of the reads cluster mainly into two of these four groups. They either cluster to WT/H1-CGR/H2 or WT/H2-CGR/H1 (See Supplementary Fig. 17 for an example shown for *sample* CT4). This supports that the two independent procedures of labeling the reads, i.e., haplotyping via small variants and the breakpoint analysis with split reads, are agreeing. Thus, we leveraged haplotyping using breakpoints.

Now, for each phase set, it has to be determined, which haplotype (H1 or H2) represents the rearranged allele. In many cases, the majority of reads votes for one scenario (e.g., WT/H1-CGR/H2). However, few reads may provide evidence for the opposite scenario (e.g., WT/H2-CGR/H1), leading to a conflicting situation. Conflicting reads might occur due to errors in haplotyping, mapping, or deviations from the assumptions that all breakpoints originate from the same haplotype.

In case of conflicting reads, the scenario with the majority of votes was selected. After these steps, all phase sets were labeled CGR and WT, respectively.

#### Expanding haplotyping to a set of chromosomes

The assumption here is that the chromosomal rearrangements originate exclusively from one allele, i.e., maternal or paternal. To expand the haplotyping across chromosomes, we combine all the CGR phase sets and WT phase sets, respectively, across the affected chromosomes.

#### Phasing RNA-seq reads using phased small variants

We used phased small variants for haplotyping of RNA-seq reads. To phase the RNA-seq, we used the variants located in gene bodies. We tagged the RNA-seq reads sampled from CGR or WT chromosomes. This step is implemented to be parallelized on samples and chromosomes. For that, we implemented a tool that provides *a* BAM file with tagged RNA-seq, a table providing the variants carried by genes and the read count for CGR or WT variants. We filtered *out* genes with less than 16 reads coverage on phased heterozygous variants.

### Analysis of breakpoint signature

For each breakpoint, Illumina reads with a minimum of 10 soft-clipped bases and a mapping quality ≥20 that are located ≤10 bp away from the called location were fetched using the python package pysam (version 0.15.2). The reads were separated into left (L) and right (R) depending on the distance of their alignment to the breakpoint location. If no supporting clipped reads were found in one or both directions, the window was iteratively extended up to 10 kb. The genomic distance between the median clipped-positions of the L- and R-read-groups was computed, representing the amount of gained or lost material i.e., the InDel size at hg19 aligned breakpoints. Only breakpoints supported by 3 or more reads clipped at the same reference position for each side were included in the InDel analysis. All others are specified as NA. To compute the junction homology, the 50 bp consensus sequence of L/R-reads around the respective median clipped position was aligned to the 25 bp aligned consensus sequences of the L/R-reads at the target breakpoint. The alignment was performed using the pairwise2.align.localms function of the python package biopython (version 1.73), assigning a +2 for a match, −1 for mismatch or opening a new gap, and −0.1 for extending an existing gap. These sequences were chosen with respect to the individual breakpoint’s strand annotation. For example: Considering a breakpoint with a “++”-annotation, the 50 bp consensus sequence of all L-reads around the median clipped position (i.e., 25 bp aligned sequence and 25 bp clipped) was aligned to the 25 bp aligned consensus sequence of all R-reads at the target breakpoint. A perfect alignment (score = 100) with start position 26 i.e., the start of the clipped sequence, was regarded as a blunt end breakpoint with no homology. Any perfect alignment with a shifted start position indicated a stretch of homology-based on the difference between the expected and observed alignment start position. Imperfect alignments were investigated for template-independent insertions by aligning 50 bp clipped consensus sequence of the initial breakpoint’s L/R-reads to 25 bp aligned consensus sequence of the target breakpoint. The final homology was defined as the total length of junction homology and template-independent insertions. Finally, each breakpoint was manually investigated to confirm the computed homology, discarding breakpoints with questionable L- or R-support.

### Statistical testing with empirical background models

The overall fractions of genomic features, shown as horizontal lines (expected value) in the Fig. 3 and Supplementary Fig. 4, were computed as the fraction of the genomic feature with respect to the length of all chromosomes, excluding gaps of the reference genome in the computation.

We implemented empirical background models to evaluate the association of breakpoints with features of the chromatin organization, repeats, as well as the enrichment of allelic imbalance genes proximal to breakpoints. For each type of background model, 1000 random configurations were generated. The *P*-value for enrichment was computed as the fraction of random configurations, which achieved a value greater or equal to than obtained by the original data. *P*-values were derived by aggregating the information from all cases, except for the analysis of allelic imbalance genes, where CT2 was excluded due to the high overall level of allelic imbalance genes. In the following, the different background models are explained in more detail.

1. Novel adjacencies with random coordinates: For each set of novel adjacencies, random sets were generated by shifting the coordinates. All coordinates on the same chromosome were shifted by the same random offset. In case a coordinate was shifted outside of the chromosome, it was inserted again at the other side of the chromosome. Thus, the relative distances between breakpoints were maintained, except for coordinates, which had to be inserted again. Configurations, in which at least one coordinate overlapped a region marked as the gap in the reference genome, were not considered. This type of random model was used to evaluate the enrichment of breakpoints in TAD boundaries, A/B compartments, LADs, repeat regions, and the analysis of allelic imbalance genes in the proximity of breakpoints.
2. Novel adjacencies with random connections: For the set of novel adjacencies from all cases, the connection between anchor points were permuted. Thus, the coordinates stayed the same, but the connection between breakpoints was altered. This permutation was used to evaluate A/B-compartment fusions and LAD/None-LAD fusions.
3. As an alternative to (1) for the analysis of allelic imbalance genes in the proximity of breakpoints, we implemented a permutation of the gene expression values and permuted the assignment of expression values to genes while the coordinates of the breakpoints were not changed.

### Manual curation of novel adjacencies

In a manual curation step, novel adjacencies candidates from PacBio and Illumina-based SV calls were checked for Hi-C patterns of rearrangements in Juicebox^43^. SV calls that appeared isolated and unrelated to the complex rearrangements were not considered. In case PacBio and Illumina-based novel-adjacency candidates were overlapping or very close to each other, the Illumina-based novel adjacency was used for downstream analysis when it was labeled as ‘precise’ by Delly. Otherwise, the PacBio-based call was selected. Besides the coordinates, the strand information was also considered in the curation process, when possible.

Sometimes, a novel-adjacency call was also selected, without having clear evidence in Hi-C, when it was fitting to novel adjacencies in the proximity that had Hi-C support. This was especially the case in complex regions, such as occurring in CT4 and CT5, showing a high degree of shattering and local clustering of novel adjacencies. Here, novel-adjacency calls were also selected without having prominent Hi-C support, when they were localized in a shattered region. Also, in case the strand information was not matching the Hi-C pattern, it was, in some cases, selected with reservation. However, novel adjacencies were removed again, when they led to ambiguous edges in the reconstruction graph or to connected chromosomal components, which were not compatible with Hi-C later. For the removal of conflicting novel adjacencies, those were preferred, which had low support in Hi-C.

For case CT2, one chromosomal fragment was broken up manually in the reconstruction, because it led to the connection of chromosomal components, which were not compatible with the Hi-C grid (See Section reconstruction). The position of the breakpoint was approximated from Hi-C alone.

### Generating scaffolds and derivative chromosomes

Manually curated large-scale novel adjacencies were used for the reconstruction of derivative chromosomes. Additionally, we added *a* few small-scale novel-adjacency calls (1–100 kb between the anchor points), when they were matching the anchor points of the curated large-scale novel adjacencies. We added the following numbers of small-scale novel adjacencies per case (CP2: 1, CT2: 1, CT3: 1, CT4: 3, CT5: 8, CT6: 2, CT7: 4) for the reconstruction.

In the first step, the breakpoints in the combined set were simplified, such that complementary breakpoints from different novel adjacencies, with almost identical coordinates, were adjusted to fit exactly. This shifting of breakpoints was done for distances below 50 bp and reduced the number of resulting fragments to avoid very small fragments. The resulting fragments can be connected at the start and at the end, except for telomeric fragments, which only have one connection possibility in our model. All novel adjacencies represent edges that connect fragments. Traversing the different paths of the graph yields the order and orientation of the fragments of the derivative chromosome. However, this requires that each fragment side has a maximum of one connecting edge, i.e. all paths are nonoverlapping. In case, more than one connecting edge was found, the ambiguity was resolved manually by removing novel adjacencies from the initial call set. This was necessary for the following cases (CT2: 3 removed, CT4: 3 removed, CT5: 4 removed, CT6: 4 removed). In case, two novel adjacencies were compatible with the same fragment end, they were prioritized based on how well they were supported by Hi-C, if they were found by PacBio and Illumina-based callers and how well the strandedness agreed with the Hi-C pattern. Often, this information ruled out novel adjacencies, which initially only have been taken into the set with reservation.

In case connections are missing, only incomplete derivative chromosomes can be reconstructed. These scaffolds can be grouped further based on shared Hi-C signal.

### Grouping scaffolds to derivative chromosomes

The reconstruction graph is sometimes incomplete for individual derivative chromosomes of complex cases (Fig. 2d), such that traversing the graph does not result in complete reconstructions. However, often still larger components, i.e., scaffolds can be derived. Afterward, these scaffolds can be grouped together by Hi-C based on the ectopic interactions between different scaffolds. For this task, we span a two-dimensional grid across the Hi-C map. The grid lines are defined by the breakpoint coordinates. The grid cells can be checked for ectopic interactions. The assumption is that fragments pairs with ectopic interactions are located in the same derivative chromosome. However, the other way around the assumption is not valid, because chromosomal fragments, which do not share any ectopic contacts, are not necessarily in different derivative chromosomes. The absence of ectopic interactions could as well result from large genomic distances between the fragments or the occurrence of different arms in the derivative chromosome. Nevertheless, the first rule is powerful enough to group almost all of the non-completed components into derivative chromosomes. Even though the order in the group is not necessarily clear, they still belong to the same derivative chromosome. The described approach can only work when the same chromosomal fragment is not part of different derivate chromosomes, which could happen in case of duplications or other copy number gains. In this case, the assumption that groups from different derivative chromosomes have mutually exclusive ectopic interaction patterns would be violated.

### Recomposing Hi-C maps of reconstructed derivative chromosomes

The reconstruction of Hi-C maps was done based on the original Hi-C maps created for reference genome hg19. According to the order of the chromosomal fragments in the derivative chromosomes, the corresponding parts of the Hi-C map were extracted using the straw-library^43^ and composed. In case the chromosomal fragment appeared in an inverted orientation, the extracted part of the original Hi-C map was inverted as well. In contrast to breakpoint coordinates, which are in base-pair resolution, the Hi-C map consists of larger bins. Therefore, the starts of chromosomal fragments were rounded up to the start of the next bin, and the end of the fragments was rounded down to the closest end of the bin. If the resulting piece of the Hi-C map was smaller than one bin, the chromosomal fragment was ignored in the reconstruction, thus small fragments could not be considered in the reconstruction.

The Hi-C map is an overlay of the signal from the WT allele and the rearranged allele. By the process of recomposing the Hi-C map, the Hi-C patterns originating from the rearranged allele are brought into order. However, at the same time, the Hi-C patterns from the WT allele become reshuffled, resulting in rearrangements patterns as observed before for the mutated allele. To mitigate artifacts from the WT allele caused by the recomposition, we subtracted from the recomposed map a control WT map, which was recomposed in the same manner. As the WT allele is expected to contribute roughly half of the signal in the Hi-C map, we scaled the control map to have 50% percent of the overall signal of the sample map. The main diagonal was excluded for the computation of the scaling factor. The aim of the subtraction is to enrich the signal from the rearranged allele, even it is not working perfectly, and artifacts may remain the in the map.

### Permutation of reconstructed scaffolds to complete reconstructions

For some cases, it was only possible to reconstruct larger scaffolds that were not complete yet. However, by the use of Hi-C, the scaffolds could be grouped and assigned to the derivative chromosome they belong to. Within these groups, the order and orientation of scaffold is unknown, because no direct sequencing-based support for a connection between the scaffolds is available. In this case, we applied a permutation-based approach to propose a draft of the derivative chromosome.

In case two scaffolds were grouped together, and both of them have a telomeric end, the scaffold was connected in the only possible manner. In case three or more scaffolds were grouped into a derivative chromosome, the two scaffolds with a telomere defined the ends of the derivative chromosomes, and for the remaining scaffolds, all possible orders and orientations were enumerated. By this, (*N* − 2)! * 2^(*N*−2)^ different combinations had to be tried out, where *N* is the number of scaffolds. This strategy was applied for groups of scaffolds with exactly two telomeric ends and group sizes >2. We limited so far the reconstruction to five components, i.e., two telomeric fragments and three middle parts. It is noted, that some scenarios, such as appending fragments to a telomeric end or the presence of duplicated genomic regions^19^, were not considered in the current implementation.

In order to assess the plausibility of each solution, we visualized the corresponding recomposed Hi-C maps (See section ‘Recomposing Hi-C maps’) and evaluated the visualizations manually. A recomposed Hi-C map, which does not show any rearrangements, is assumed to indicate a proper reconstruction, and only one of the permutations should represent the correct or at least the best solution. An exception is recomposed Hi-C maps with very small scaffolds that contain only very small pieces of the Hi-C map, in the most extreme case, only one bin. Here, the differences between different solutions can be absent (1 bin) or very small, making the distinction of solutions difficult or impossible.

Additional to the manual inspection, we computed a simple score for each of the permutations. The associated recomposed Hi-C map can be separated into tiles (See Fig. 5), according to the underlying chromosomal fragments. We computed the score as the sum over all tiles defined by all non-redundant pairwise combinations of fragments.

The area of a tile in the Hi-C map is defined by the two fragments, start *s* and end *e*, describing the start bin and end bin of the corresponding fragment in the recomposed Hi-C map *m*. The subscore of a tile is computed by weighting the Hi-C signal at position *ij* with the distance of the pixel to the main diagonal |*i* − *j* | . These weighted Hi-C values are summed up in the four corners of the tile:

1 $${{{{{{\mathrm{subscore}}}}}}}\left({s}_{1},{e}_{1},{s}_{2},{e}_{2}\right)=	 \mathop{\sum }\limits_{i={s}_{1}}^{{s}_{1}+w-1}\mathop{\sum }\limits_{j={s}_{2}}^{{s}_{2}+w-1}\left|i-j\right|\cdot {m}_{{ij}}+\mathop{\sum }\limits_{i={s}_{1}}^{{s}_{1}+w-1}\mathop{\sum }\limits_{j={e}_{2}-w+1}^{{e}_{2}}\left|i-j\right|\\ 	 \cdot {m}_{{ij}}+\mathop{\sum }\limits_{i={e}_{1}-w+1}^{{e}_{1}}\mathop{\sum }\limits_{j={s}_{2}}^{{s}_{2}+w-1}\left|i-j\right|\cdot {m}_{{ij}}+\mathop{\sum }\limits_{i={e}_{1}-w+1}^{{e}_{2}}\mathop{\sum }\limits_{j={e}_{2}-w+1}^{{e}_{2}}\left|i-j\right|\cdot {m}_{{ij}}$$

The window size *w*, was set to 5, or to (*e* − *s* + 1) in case the corresponding dimension of the tile was smaller than 5.

The rational of the score is that the parts of the Hi-C map with the largest values should be at the main diagonal or close by. In case of an erroneous reconstruction, high-intensity values will occur further away from the main diagonal, because reconstruction error should appear as rearrangement. Thus, the working assumption is that errors in the reconstruction lead to an increase in the score. For our cases, the solution with the lowest score was identical to the solution selected by the manual inspection.

### Evaluation of the reconstructions of derivative chromosomes

We performed several evaluations of our reconstruction. We generated custom genomes as Fasta files for each case according to the respective reconstruction of derivative chromosomes. In the next step, we aligned PacBio long-reads to the corresponding custom genome and checked, how many reads spanned the junctions in a window of 100 bp around the junction Supplementary Fig. 19b. We also mapped the Hi-C data to custom genomes and inspected the Hi-C maps. The Hi-C data is more difficult to evaluate because the map is an overlay of the rearranged allele, which is now ideally in order, but the WT allele produces patterns of rearrangements. We tried to mitigate the effect of the WT allele, by subtracting a control sample which was scaled to 50% of the overall intensity on autosomes (neglecting the diagonal and first subdiagonal). This operation reduced the intensity, but some patterns remained Supplementary Data 2.

### Additional software tools

BioRender (https://biorender.com/) was used to create schematics of cells in Fig. 1b. The Circlize package^51^ was used to create circus plots. Color palettes were created with https://colorbrewer2.org and the Paletton online tool (https://paletton.com). The read coverage of Illumina GS reads for chromosomal fragments was computed using Bioconductor package bamsignals (https://bioconductor.org/packages/release/bioc/html/bamsignals.html). Bioconductor package GenomicRanges^52^ was used to compute distances and overlaps between genomic intervals.

### Reporting summary

Further information on research design is available in the Nature Research Reporting Summary linked to this article.

## Supplementary information

Supplementary Information

Supplementary Data 1

Supplementary Data 2

Reporting Summary

## Data Availability

The data that support this study are available from the corresponding authors upon reasonable request. Informed consents from patients do not cover the deposition of sequencing data from the patient samples. These data are available only upon request from S.M. (stefan.mundlos@charite.de). Data can be shared for research purposes with permission of the patient or his/her legal guardian. The gene annotation from Gencode (v19) was used. TAD annotations for hg19 were downloaded from the website of the 3D genome browser for cell line GM12878^53^. A/B-compartment annotation was taken from previously published work^25^ (GEO GSE63525), the subcompartments were collapsed to A and B compartments. LAD annotation for T cells was taken from previously published work^26^ (GEO GSE94971). A list of housekeeping genes was taken from previously published work^54^. Repeats were downloaded from UCSC genome browser^55^ for hg19 via the table browser^56^ (group: Repeats, track: RepeatMasker, table: rmsk).

## Code availability

Code for the reconstruction approach: https://github.molgen.mpg.de/schoepfl/chromosome_reconstruction. Code for haplotyping and RNA-seq phasing: https://github.com/moeinzadeh/Chromothripsis_haplotyping.
