# Supplementary material for: Integration of Hi-C with short and long-read genome sequencing reveals the structure of germline rearranged genomes
Source: Nat Commun. 2022 Oct 29;13:6470. doi: 10.1038/s41467-022-34053-7 (PMC9617858; doi:10.1038/s41467-022-34053-7)
Supplement: Supplementary file 1 — Supplementary Information [file 41467_2022_34053_MOESM1_ESM.pdf]

# Supplementary Information

## Integration of Hi-C with short and long-read genome sequencing reveals the structure of germline rearranged genomes

Schöpflin, Melo et al.

### Supplementary Figure 1

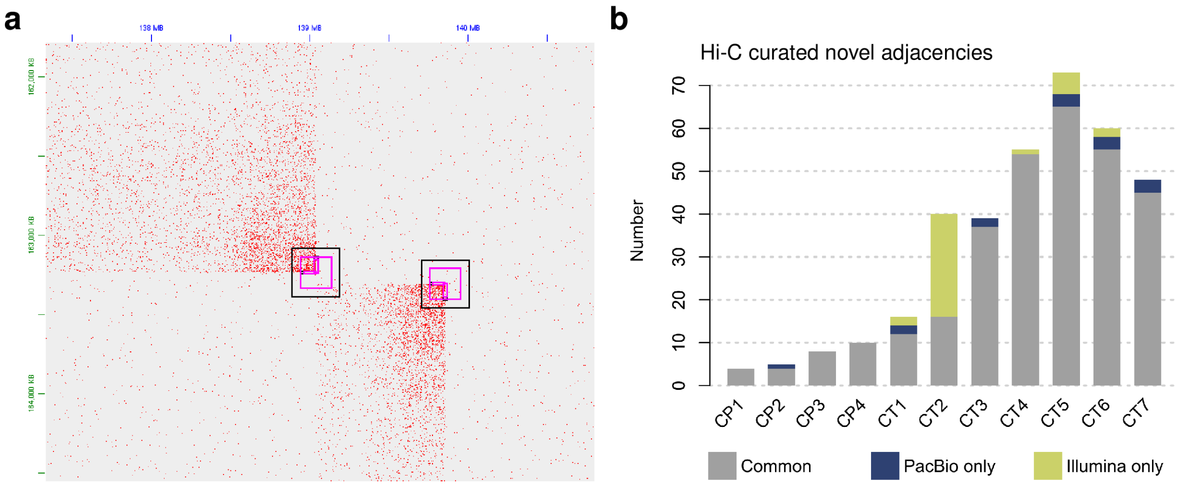


**Supplementary Figure 1**: Comparison of Illumina and PacBio-based identification of novel adjacencies. (**a**) Overlay of Illumina based calls (pink) and PacBio-based calls (black) in Juicebox[1]. The center of the square represents the novel adjacency coordinates. The angle within the square indicates the strand orientations of the novel adjacency, which recapitulates here the sector of the ectopic Hi-C pattern. (**b**) Number of large-scale novel-adjacencies, which had evidence in Hi-C and were detected by one or both of the GS based methods. Please note, that the initial filtering of PacBio based and Illumina based calls is different.

**Supplementary Figure 2**

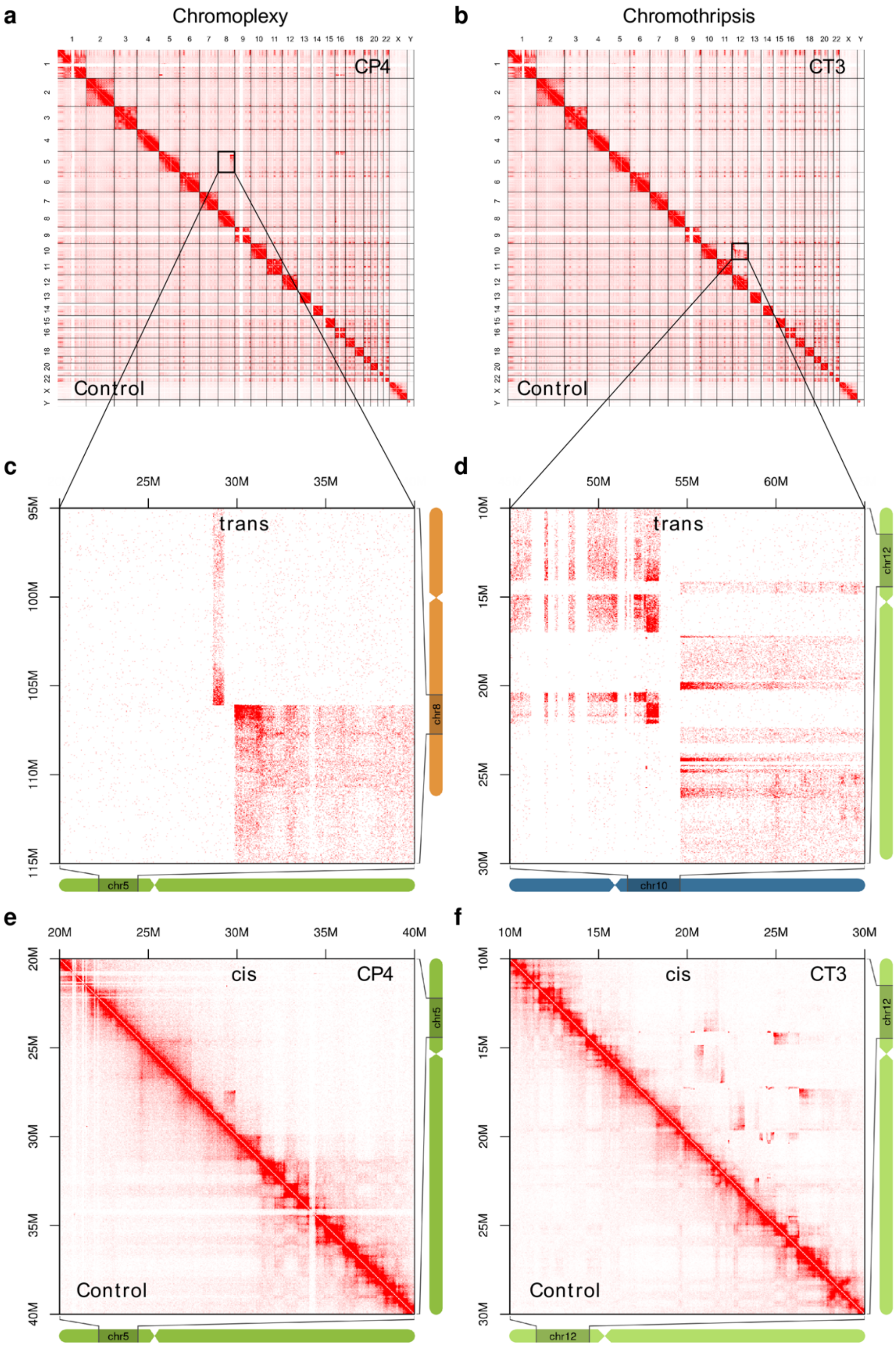


**Supplementary Figure 2**: Chromoplexy (left) and chromothripsis cases (right) show different Hi-C patterns at different zoom levels. **a,b**) Genome-wide Hi-C maps **c,d**) Zoom-in of trans Hi-C maps showing ectopic contacts between chr5 and chr8, and between chr10 and chr12, respectively. The chromosome schematics at the right and bottom indicate the regions of the chromosome, which are displayed. **e,f**) Zoom-in of cis Hi-C maps of chr2 and chr5, respectively. While the chromoplexy cases show fewer rearrangements, which occur often between chromosomes, the chromothripsis cases exhibit more and more complex rearrangements. The breakpoints occur often in clusters and the rearrangements are intermingled leading to complex ectopic Hi-C patterns in cis (F) and trans (D).

## Supplementary Figure 3

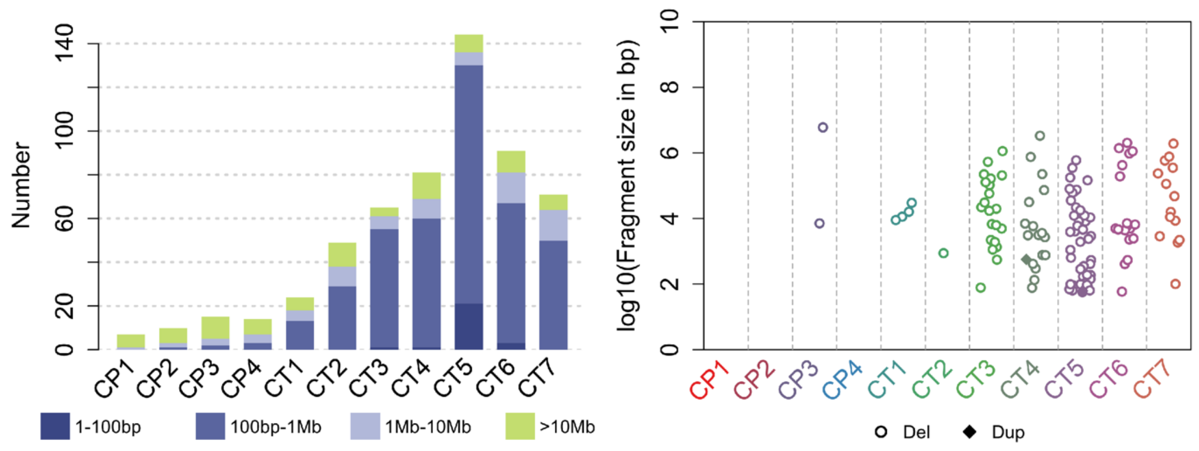


**Supplementary Figure 3**: Size of chromosomal fragments and analysis of their short-read coverage. (**a**) Size of chromosomal fragments in chromoplexy (CP1-CP4) and chromothripsis-like (CT1-CT7) cases, which emerge upon the breakage of chromosomes. (**b**) Short-read coverage analysis measuring the read coverage with respect to control samples for each of the inferred chromosomal fragments to detect copy number alterations. A (median) ratio smaller than 0.7 per fragment was considered to be a deleted fragment, a ratio above 1.3 was considered as duplication/amplification. Only fragments with unusual coverage ratio are shown here with their corresponding size for the individual cases.

## Supplementary Figure 4

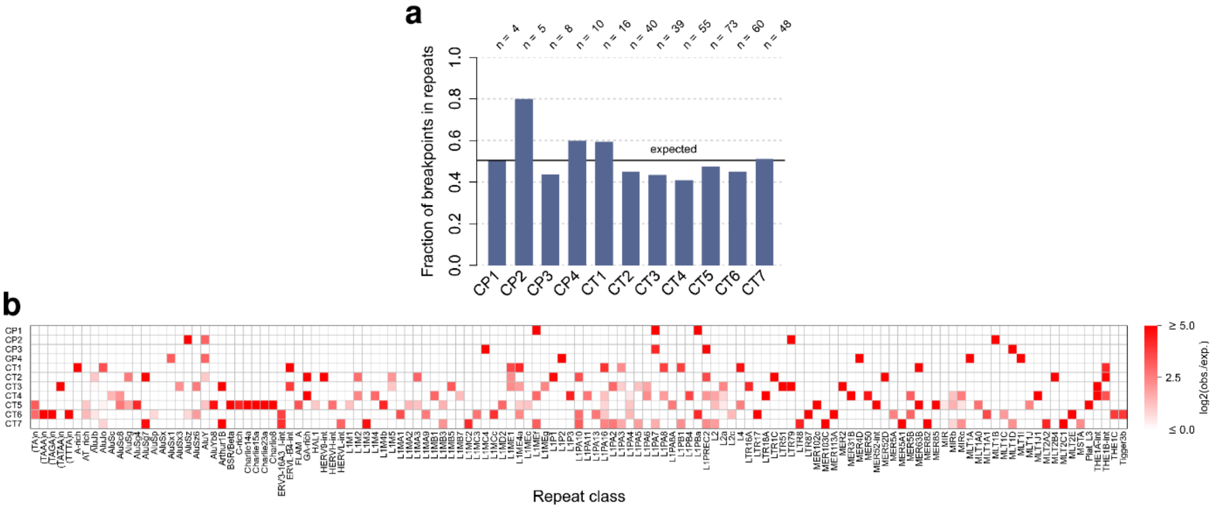


**Supplementary Figure 4**: Overlap of breakpoints with repeats. (**a**) The fraction of breakpoints, which is located in repeats. *N* is the number of novel adjacencies. The black horizontal line indicates the genome wide fraction of repeats in repeat masker annotations from UCSC for autosomes and chrX (excluding reference gaps). (**b**) Enrichment analysis for different repeat classes: log2 enrichment for all repeat classes, which are overlapping with a breakpoint in at least one sample. Note, negative values (depletions) are not shown.

## Supplementary Figure 5

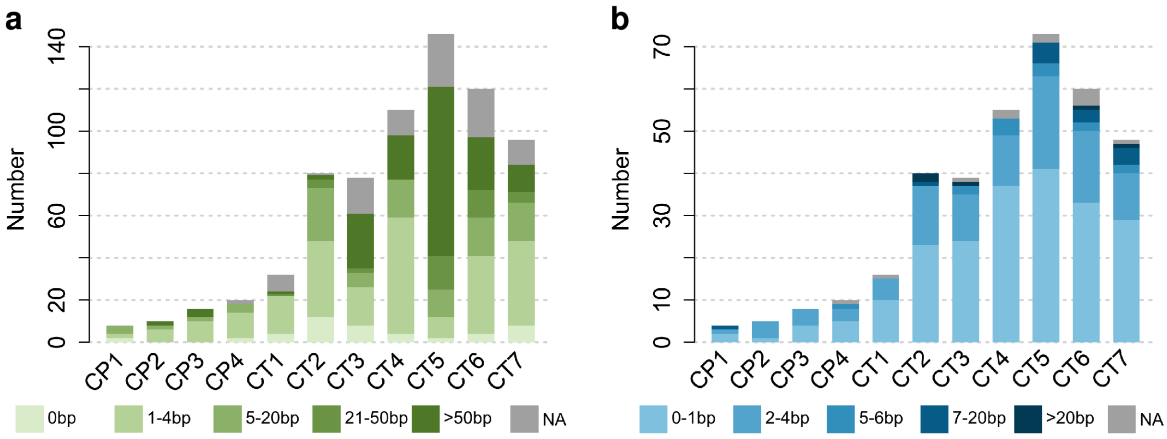


**Supplementary Figure 5**: InDels and microhomology at breakpoints. (**a**) Number and size of InDels at novel adjacency breakpoints. (**b**) Number and size of microhomologies at novel adjacency junctions.

**Supplementary Figure 6**

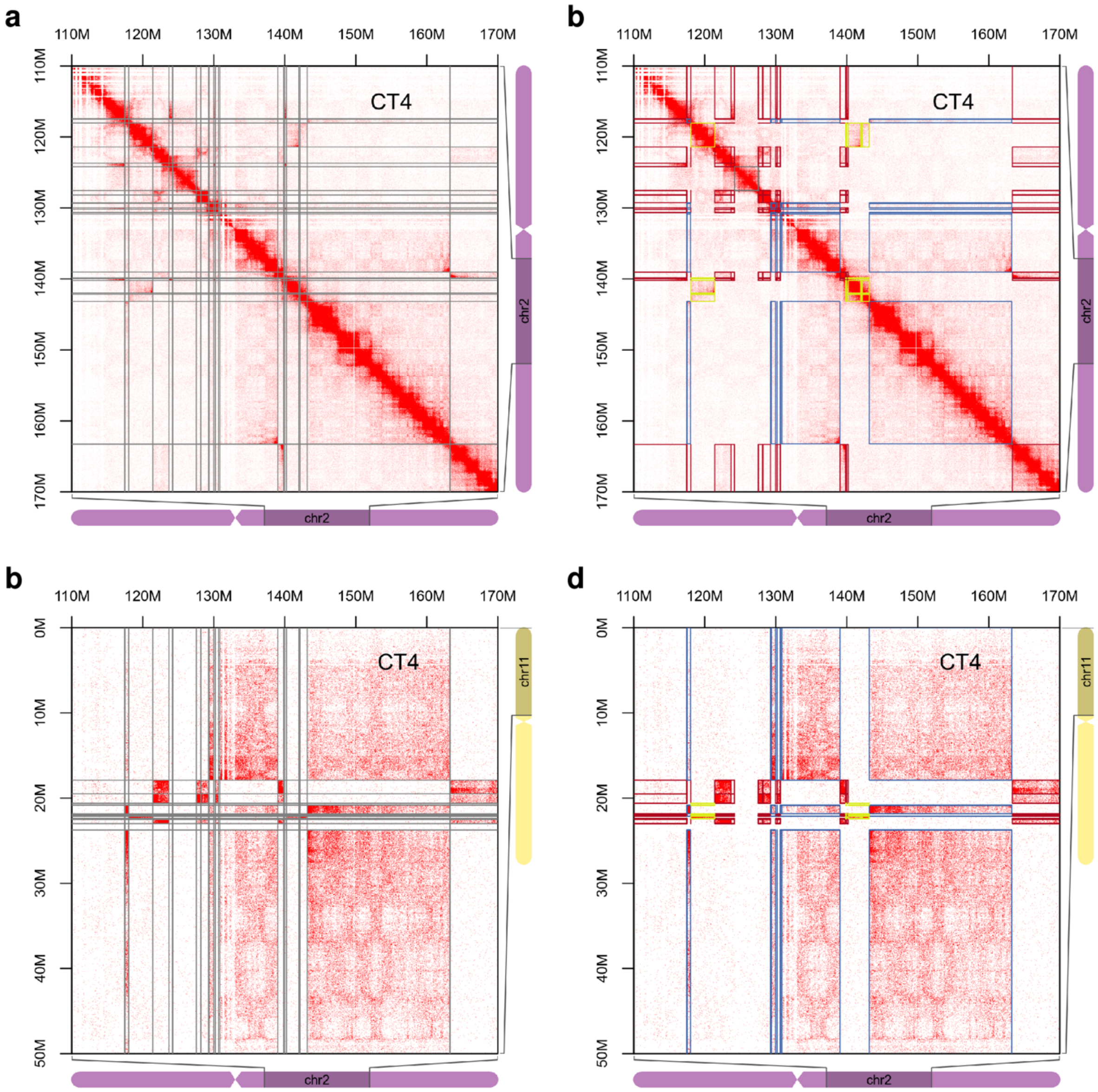


**Supplementary Figure 6**: Visualization of a grid, which is defined by the breakpoints of the chromosomal fragments. (**a**) Each breakpoint defines a vertical and a horizontal line in the grid projected on the cis Hi-C map. Ectopic Hi-C patterns in a grid cell indicate that both fragments are located in the same derivative chromosome. (**b**) After the reconstruction of derivative chromosomes, the components of the derivative chromosomes can be overlayed with the Hi-C map. Colored boxes represent the putative area for interactions between chromosomal fragments, which are part of the same derivative chromosome. This illustrates the intermingling of ectopic Hi-C patterns from different derivative chromosomes (red, blue and yellow rectangles) in the Hi-C map. (**c**) Same as (a), but for a trans Hi-C map. (**d**) Same as (b), but for a trans Hi-C map. When no fragments are shared between different derivative chromosomes (i.e. by copy number gains), the different sets (red, blue, yellow) are mutually exclusive.

**Supplementary Figure 7**

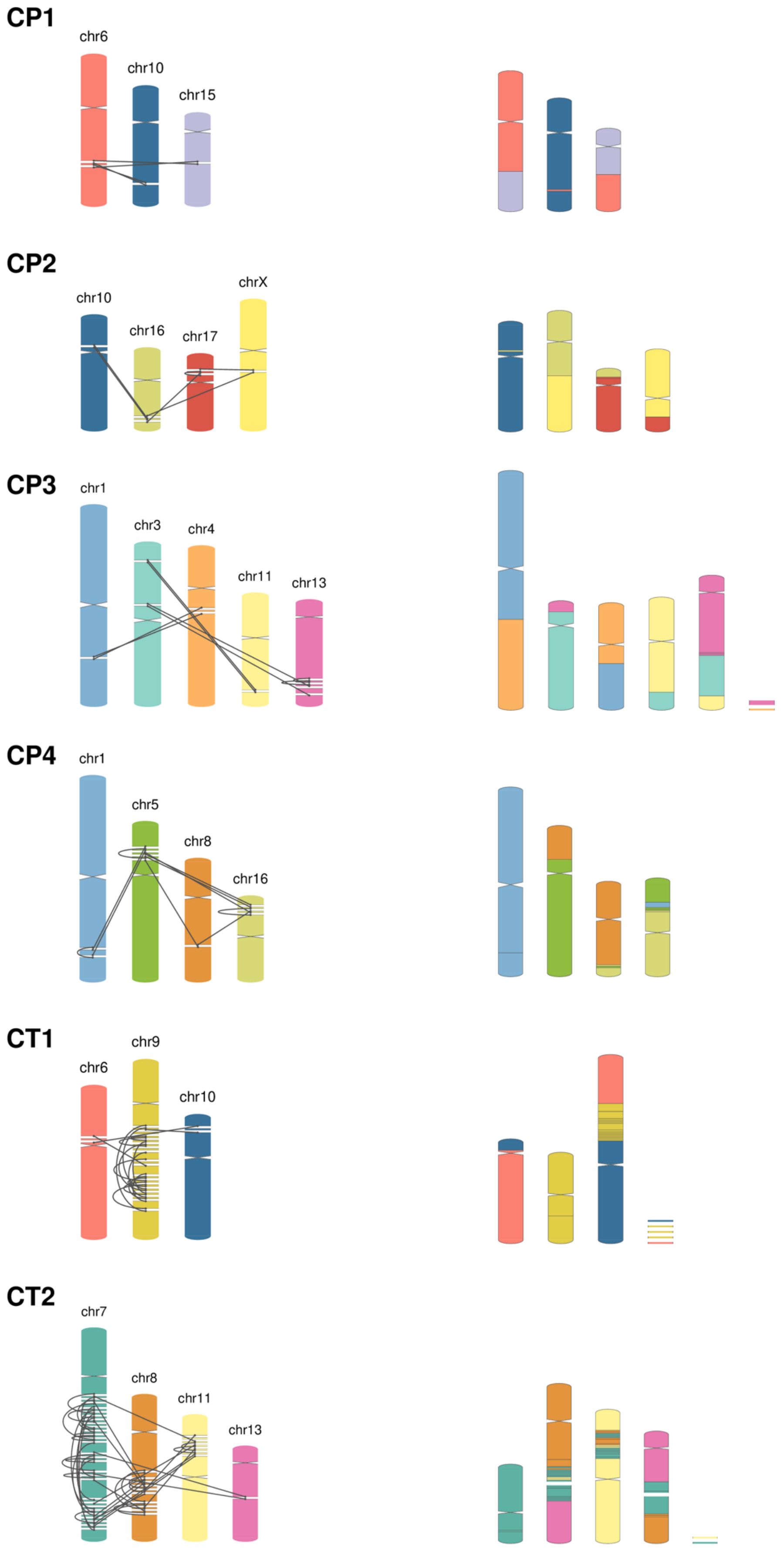

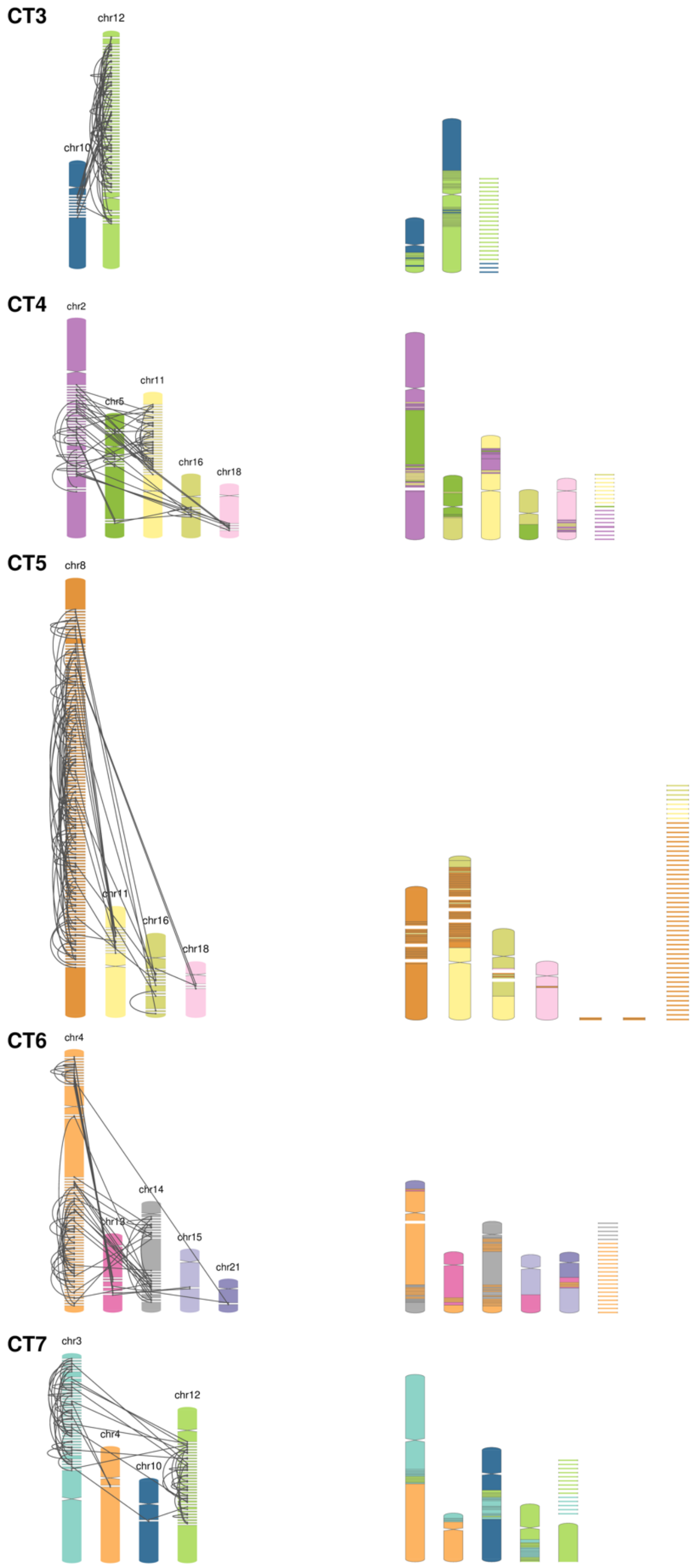


**Supplementary Figure 7**: Overview of reconstructions. Left hand side: Reconstruction graphs for all 11 cases. Right hand side shows derivative chromosomes for all 11 cases, if possible as whole derivative chromosome or, if not, as scaffolds grouped to derivative chromosomes without knowing the connection between scaffolds. Leftover fragments are shown as stacks right of the derivative chromosomes. The scaffolds were ordered and oriented further by a permutation approach. Note, the size of small fragments and telomeric ends is enhanced to improve visibility. Centromeric fragment of der(8) of CT5 was too small to visualize the centromere.

**Supplementary Figure 8**

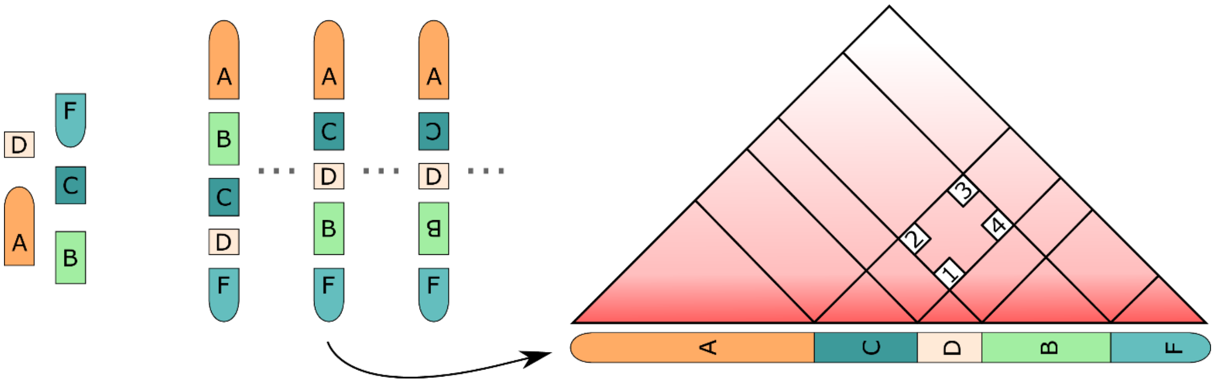


**Supplementary Figure 8**: Permutation of different solutions for derivative chromosomes and corresponding Hi-C maps. If the derivative chromosome could be resolved to the level of a few remaining scaffolds, all possible orders and orientations of scaffolds are enumerated. The Hi-C map is recomposed according to the configuration of scaffolds. For each configuration, a score is computed to evaluate recomposed Hi-C maps. For each of the resulting tiles (here shown only for tile C-B), the Hi-C signal in the corners (regions 1-4) is considered and weighted by the distances between the Hi-C coordinates on the rearranged chromosome.

## Supplementary Figure 9

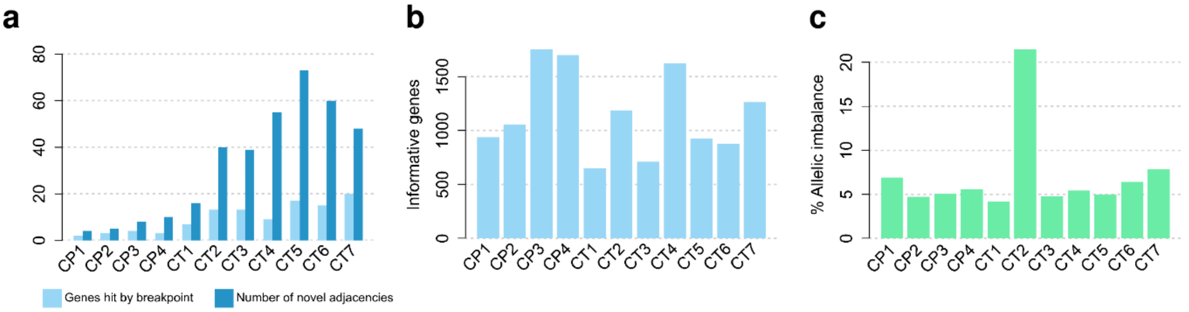


**Supplementary Figure 9**: Overview of genes and gene expression. (**a**) Number of genes with at least one breakpoint in the gene body and number of large-scale novel adjacencies. (**b**) Number of informative genes per sample. (**c**) Percentage of informative genes with allelic imbalance in gene expression.

## Supplementary Figure 10

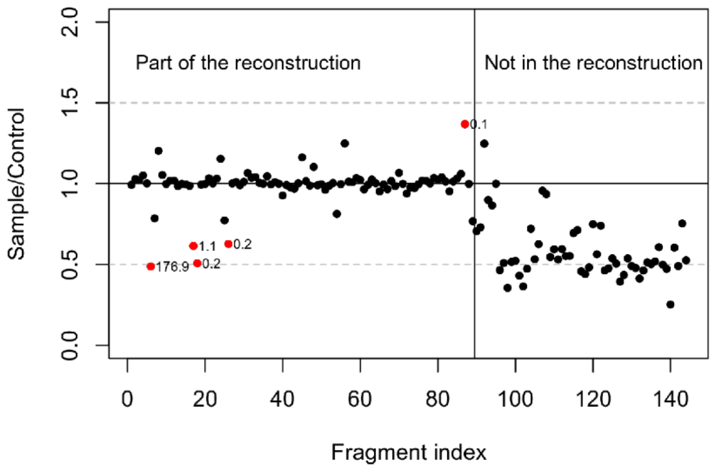


**Supplementary Figure 10** Coverage analysis for the chromosomal fragments of CT5. The median ratio of the sample with respect to control samples is shown, as well as the fragment size in kb next to the fragment dot for selected fragments. The ratio indicates that few deleted fragments are still part of the reconstruction. Many fragments, which are not part of the reconstruction, appear indeed deleted.

## Supplementary Figure 11

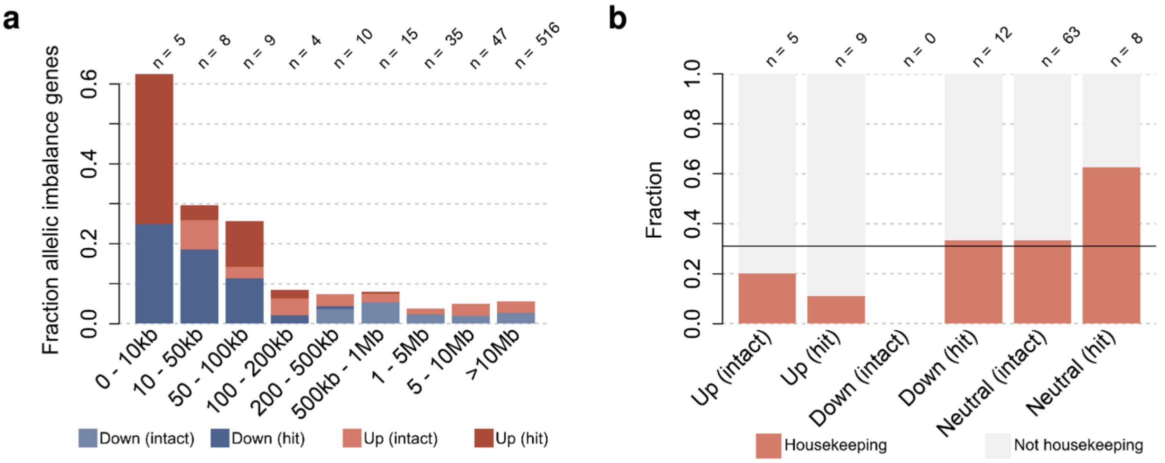


**Supplementary Figure 11**: Analysis of gene expression. (**a**) Fraction of allelic imbalance genes for different distances between the TSS and the closest breakpoint stratified into upregulated and downregulated genes, as well as genes which are hit by a breakpoint and which are intact, respectively. N shows the number AIGs per distance bin. (**b**) Fraction of housekeeping genes in the different classes of genes within 200 kb to the closest breakpoint. Genes are stratified according to being up- or down-regulated, or neutral (not regulated), as well as being intact or hit by a breakpoint.

## Supplementary Figure 12

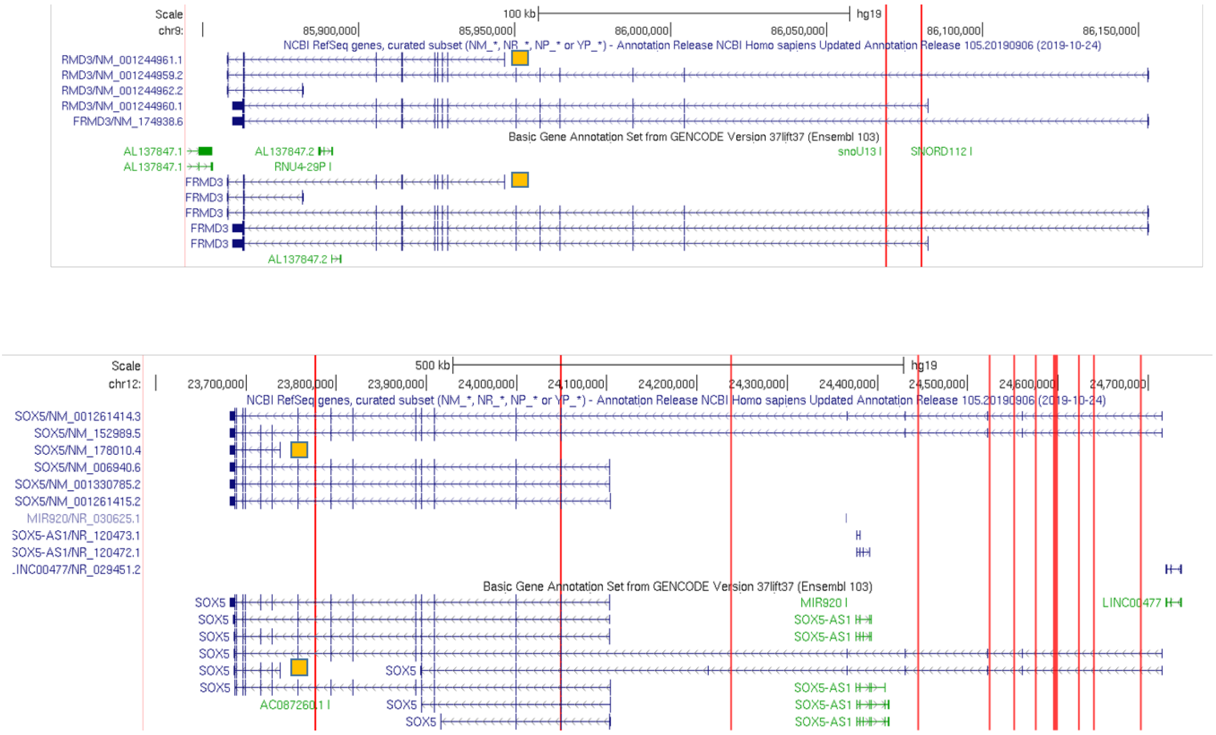


**Supplementary Figure 12**: Examples of genes, which are hit by a breakpoint (red vertical lines) and are upregulated. Transcript variants (marked by a yellow rectangle) not hit by the breakpoint exist in both cases. Visualizations of genes were created with the UCSC genome browser[2].

## Supplementary Figure 13

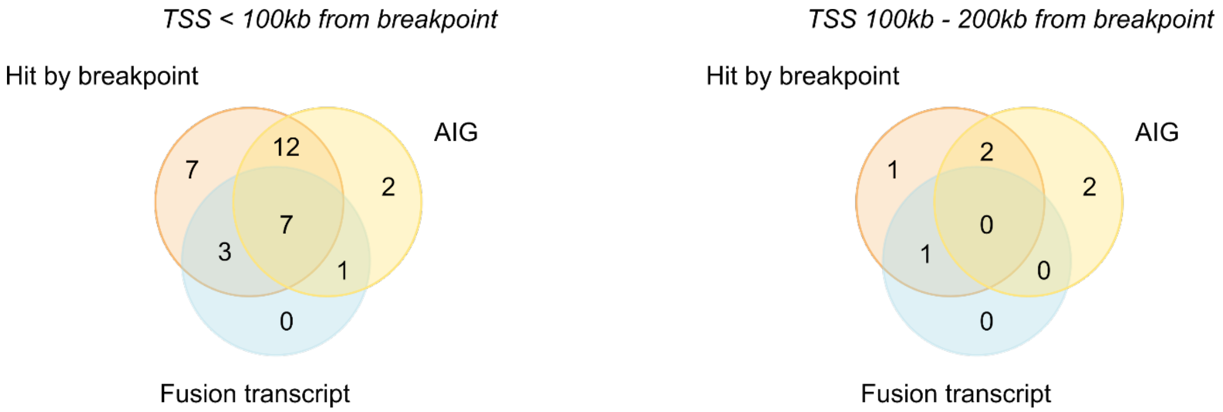


**Supplementary Figure 13**: Venn diagrams for different groups of genes. Diagrams show informative genes, which are i) hit by a breakpoint, ii) show allelic imbalance in expression, iii) are predicted to be involved in a fusion transcript for the distance <100 kb (left side) and for genes between 100-200 kb from the next breakpoint (right side).

## Supplementary Figure 14

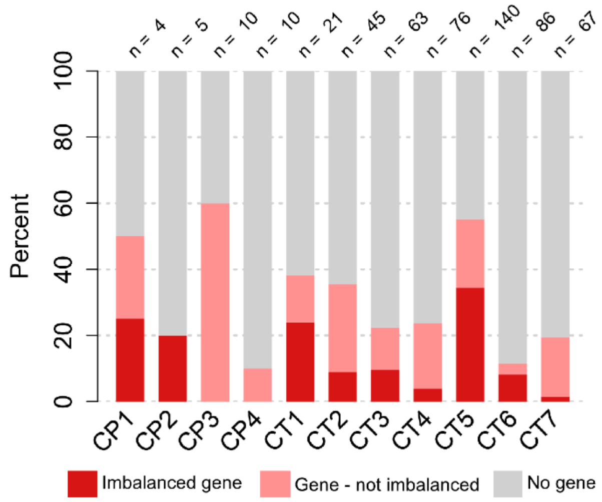


**Supplementary Figure 14**: Fraction of breakpoints with respect to informative genes in proximity. Percentage of breakpoints, with (i) at least one allelic imbalance gene in ±200 kb proximity to the breakpoint (dark red), (ii) at least one expressed and phased gene in ±200 kb proximity, but none of them is allelic imbalanced (light red), and (iii) no informative (expressed and phased) gene in ±200 kb proximity of the breakpoint at all (grey). N shows the number of breakpoint *anchors*.

## Supplementary Figure 15

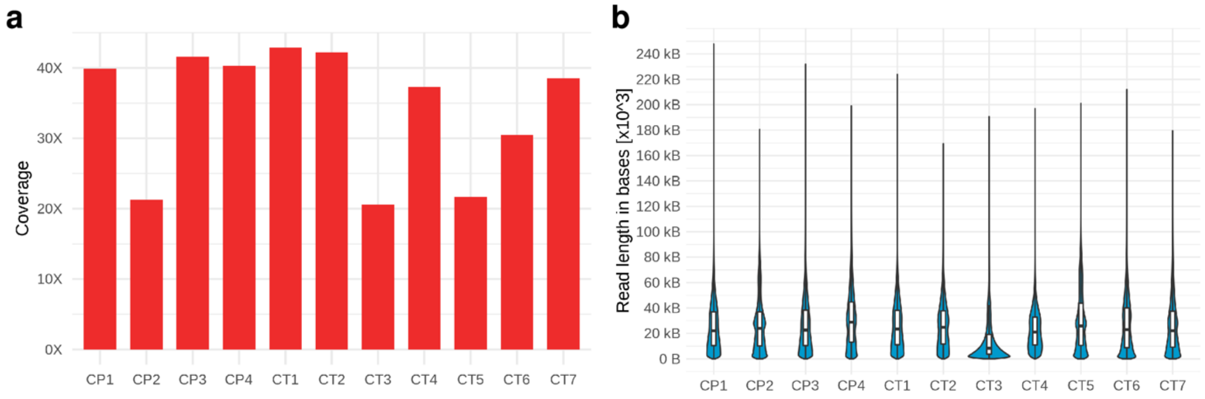


**Supplementary Figure 15**: Statistics of PacBio long read sequencing. (**a**) Mean coverage per sample. (**b**) Distribution of read length per sample. The box plots describe the median (thick horizontal line), the first and third quartiles (box) and the most extreme values (whiskers) of the distribution.

## Supplementary Figure 16

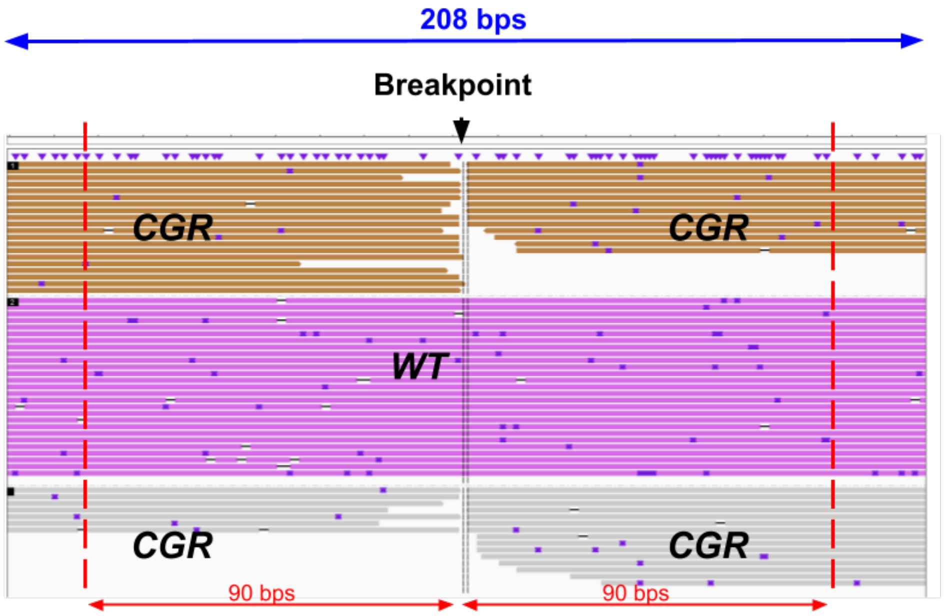


**Supplementary Figure 16**: Aligned PacBio reads visualized in genome viewer IGV[3]. Reads are colored according to the halplotypes H1 (brown), H2 (purple) and NoHapInfo (grey) from the HapCUT2 analysis. Additionally, the PacBio reads can be separated by being breakpoint-spanning (WT) or ending at breakpoints (CGR), as well as NoCGRinfo (not present here).

**Supplementary Figure 17**

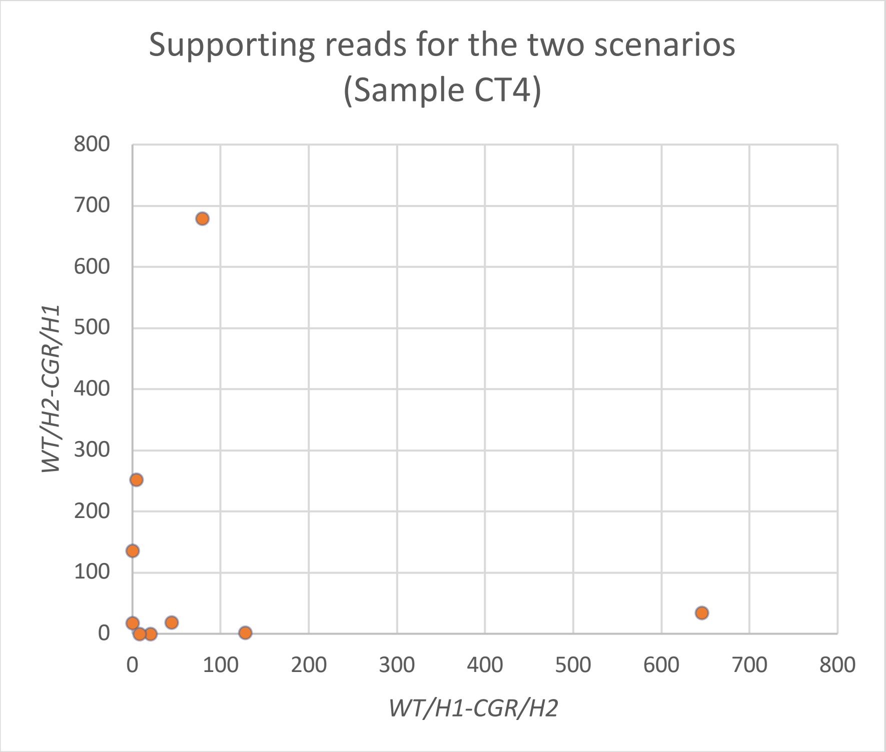


**Supplementary Figure 17:** Number of supporting reads for the two haplotype scenarios. Assignment of haplotype (H1 and H2) to the WT and rearranged (CGR) allele, respectively. X-axis: Reads supporting H1 being wildtype and H2 being the rearranged allele, y-axis: reads supporting the opposite scenario. Phase sets with clear assignments are located close to one of the axes, data points closer to the diagonal indicate conflicting reads.

## Supplementary Figure 18

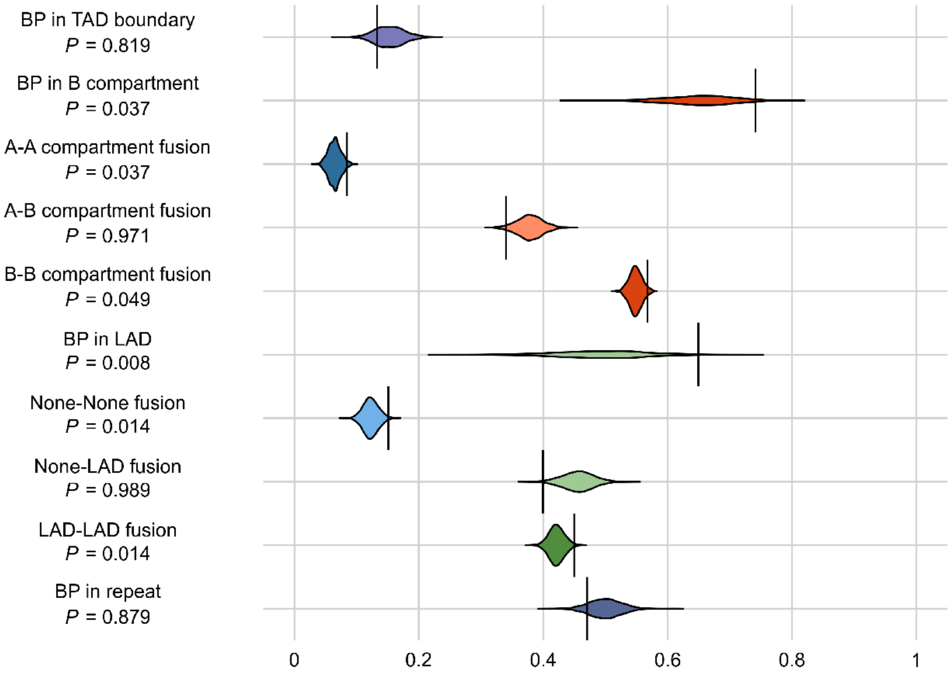


**Supplementary Figure 18**: Statistical testing for the enrichment of breakpoints of novel adjacencies with respect to features of the chromatin organization (TAD boundaries, A/B compartments, LADs) as well as repeat regions, using empirical background models. The observed value obtained from the real breakpoints pooled over the samples is shown as vertical line for every feature. The corresponding distribution of the empirical background model is shown as colored area, the associated P-value is indicated in the legend on the left-hand side. The tests were performed right-sided, adjustments for multiple testing were not performed.

**Supplementary Figure 19**

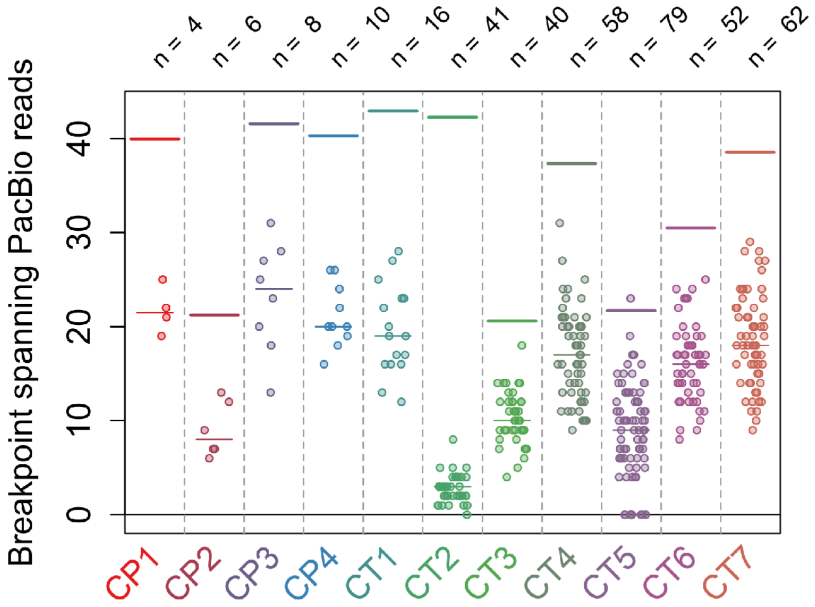


**Supplementary Figure 19:** Number of junction spanning PacBio long reads, which were mapped to custom genomes generated based on the reconstruction. Bold horizontal lines indicate the mean read coverage per sample. Thin horizontal lines indicate the median number of junction spanning reads. Junctions are defined by the novel adjacencies detected by PacBio and Illumina based SV calling, which were curated with Hi-C. Additional to the large-scale novel adjacencies, also few small-scale novel adjacencies entered the reconstruction (See methods section ‘Generating scaffolds and derivative chromosomes’).

**Supplementary Figure 20**

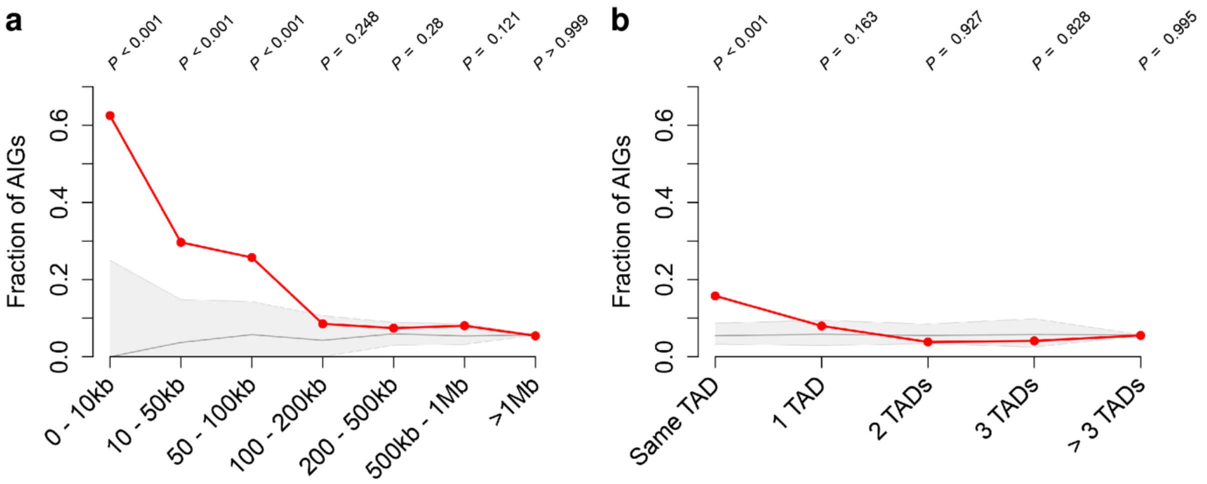


**Supplementary Figure 20**: Alternative approach to compute an empirical background model to test for enrichment of allelic imbalance genes (AIGs) close to breakpoints. Instead of generating novel adjacencies with randomly shifted coordinates, the gene expression table was permuted 1000 times (see Methods) to obtain empirical P-values. (**a**) Differential gene expression analysis around breakpoints: Fraction of allelic imbalance genes (AIGs) with respect to the distance between an expressed gene and the closest breakpoint (red line). As a control, simulations of random breakpoints were performed. The light grey area with the grey line indicates the $5^{th}$-percentile, median and $95^{th}$ percentile, respectively, expected by chance. P-values were computed by comparing each observed value against values obtained from an empirical background model. The tests were performed right-sided, adjustments for multiple testing were not performed. (**b**) Same as (a), but the distance of a gene to the next breakpoint is measured in TAD units (Same TAD, 1 TAD, etc.). P-values were computed as described in (a).

| Subjects ID | Phenotype | Karyotyping (ISCN 2016 nomenclature) | Type | Cell type | Comment on reconstruction of derivative chromosomes |
|---|---|---|---|---|---|
| **CP1** | No pathological findings | Not performed | Chromoplexy | LCLs | |
| **CP2** | Intellectual disability, spasms, dysmorphisms | 46,XX,t(X;16;17)(q13.3;q23;p11.2) | Chromoplexy | LCLs | Involvement of chr10 not reported in karyotyping |
| **CP3** | Intellectual disability | 46,XX,t(1;4)(q31;q21.27)t(3;13)(p14.1;q33) | Chromoplexy | LCLs | Involvement of chr11 not reported in karyotyping |
| **CP4** | Intellectual disability | 46,XY,t(5;16;8)(p13.1;p13.1;q22) | Chromoplexy | LCLs | Involvement of chr1 not reported in karyotyping |
| **CT1** | Global developmental delay | 46,XY,t(6;10)(p11.2;p15),ins(der(der(10);9)(p15;q13q22)+dupXq28 | Chromothripsis | LCLs | Involvement of chrX not observed in Hi-C |
| **CT2** | Möbius syndrome | 46,XY,t(7;8;11;13) | Chromothripsis | Fibroblasts | |
| **CT3** | Intellectual disability and microcephaly | 46,XY,t(10;12)(q11.2;p11.2) | Chromothripsis | LCLs | |
| **CT4** | Intellectual disability | 46,XX,ins(2;5)(q14;q12,q33),ins(2)(q24q21),t(5;16)(q33;q12),ins(11;2)(p14;q21q22),ins(11;2)(p14;q23q24),ins(18;2)(q22;q22) | Chromothripsis | LCLs | |
| **CT5** | Intellectual disability, dysmorphisms | 46,XX,t(11;16)(p21;q12.1)+der8 | Chromothripsis | LCLs | Involvement of chr18 not reported in karyotyping |
| **CT6** | Intellectual disability | 46,XX,t(4;13)(4;14)(4;21)(13;15)(13;21)(15;21) | Chromothripsis | LCLs | |
| **CT7** | Intellectual disability | 46,XY,t(3;4)(3;10)(3;12)(4;12)(10;12) | Chromothripsis | LCLs | |

**Supplementary Table 1**: Overview of the cohort.

| Sample ID | Clinical symptoms | Gene Symbol | Allelic expression | Gene associated with ID (OMIM) | Phenotype (OMIM) | ID OMIM* | Inheritance | Candidate to explain the phenotype? |
|---|---|---|---|---|---|---|---|---|
| CP4 | Intellectual disability | USP7 | None | Yes | Hao-Fountain syndrome | 616863 | AD | Potential |
| CT2 | Möbius syndrome | CCDC132 | None | Yes | Neurodevelopmental disorder with microcephaly, seizures, and neonatal cholestasis | 619685 | AR | no |
| | | CNTNAP2 | Up | Yes | Pitt-Hopkins like syndrome 1 | 610042 | AR | no |
| | | RIMS2 | Down | Yes | Cone-rod synaptic disorder syndrome, congenital nonprogressive | 618970 | AR | no |
| CT3 | Intellectual disability and microcephaly | ABCC9 | None | Yes | Intellectual disability and myopathy syndrome | 619719 | AR | no |
| | | SOX5 | Up | Yes | Lamb-Shaffer syndrome | 616803 | AD | Potential |
| | | GRIN2B | None | Yes | Intellectual developmental disorder, autosomal dominant 6, with or without seizures | 613970 | AD | Already published |
| CT5 | Intellectual disability and lower limb ectrodactyly | CTCF | None | Yes | Intellectual developmental disorder, autosomal dominant 21 | 615502 | AD | no |
| | | CA8 | None | Yes | Cerebellar ataxia and mental retardation with or without quadrupedal locomotion 3 | 613227 | AR | no |
| CT6 | Intellectual disability and craniofacial dysmorfisms | CLCN3 | None | Yes | Neurodevelopmental disorder with hypotonia and brain abnormalities | 619512 | AD | no |
| | | TENM3 | None | Yes | Microphthalmia, syndromic 15 | 615145 | AR | no |
| CT7 | Intellectual disability and hypotonia | SUMF1 | Down | Yes | Multiple sulfatase deficiency | 272200 | AR | no |
| | | NGLY1 | None | Yes | Congenital disorder of deglycosylation 1 | 615273 | AR | no |
| | | ITPR1 | None | Yes | Gillespie syndrome | 206700 | AD, AR | no |

**Supplementary Table 2**: Selected genes, for which breakpoints were identified and their putative association to the phenotype.

## Supplementary References

1. Durand NC*, et al.* Juicebox Provides a Visualization System for Hi-C Contact Maps with Unlimited Zoom. *Cell Syst* **3**, 99-101 (2016).

2. Kent WJ*, et al.* The human genome browser at UCSC. *Genome Res* **12**, 996-1006 (2002).

3. Robinson JT*, et al.* Integrative genomics viewer. *Nat Biotechnol* **29**, 24-26 (2011).
