# Supplementary figures and images for: Integration of Hi-C with short and long-read genome sequencing reveals the structure of germline rearranged genomes
Source: Nat Commun. 2022 Oct 29;13:6470. doi: 10.1038/s41467-022-34053-7 (PMC9617858; doi:10.1038/s41467-022-34053-7)

CP1

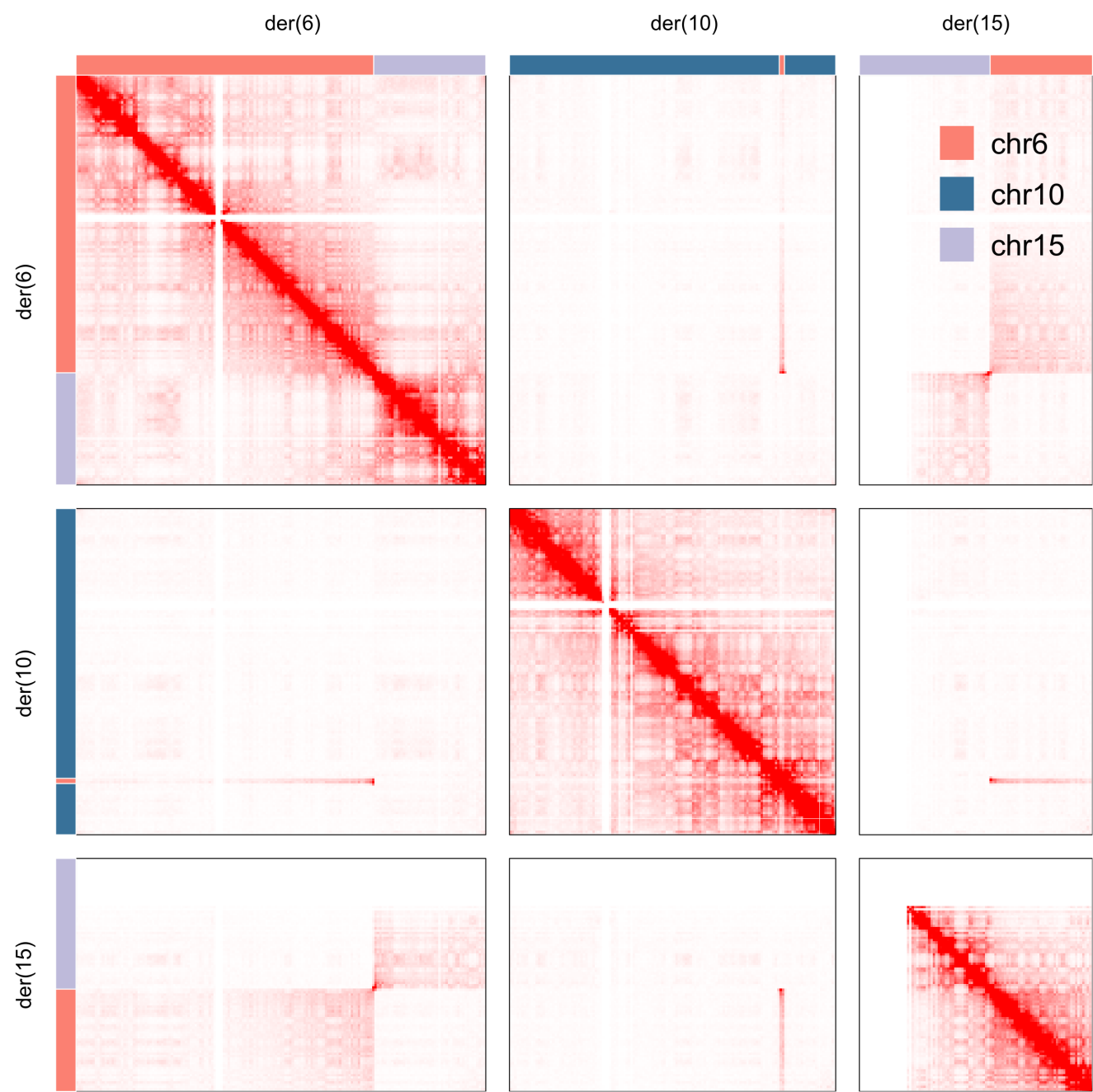

CP2

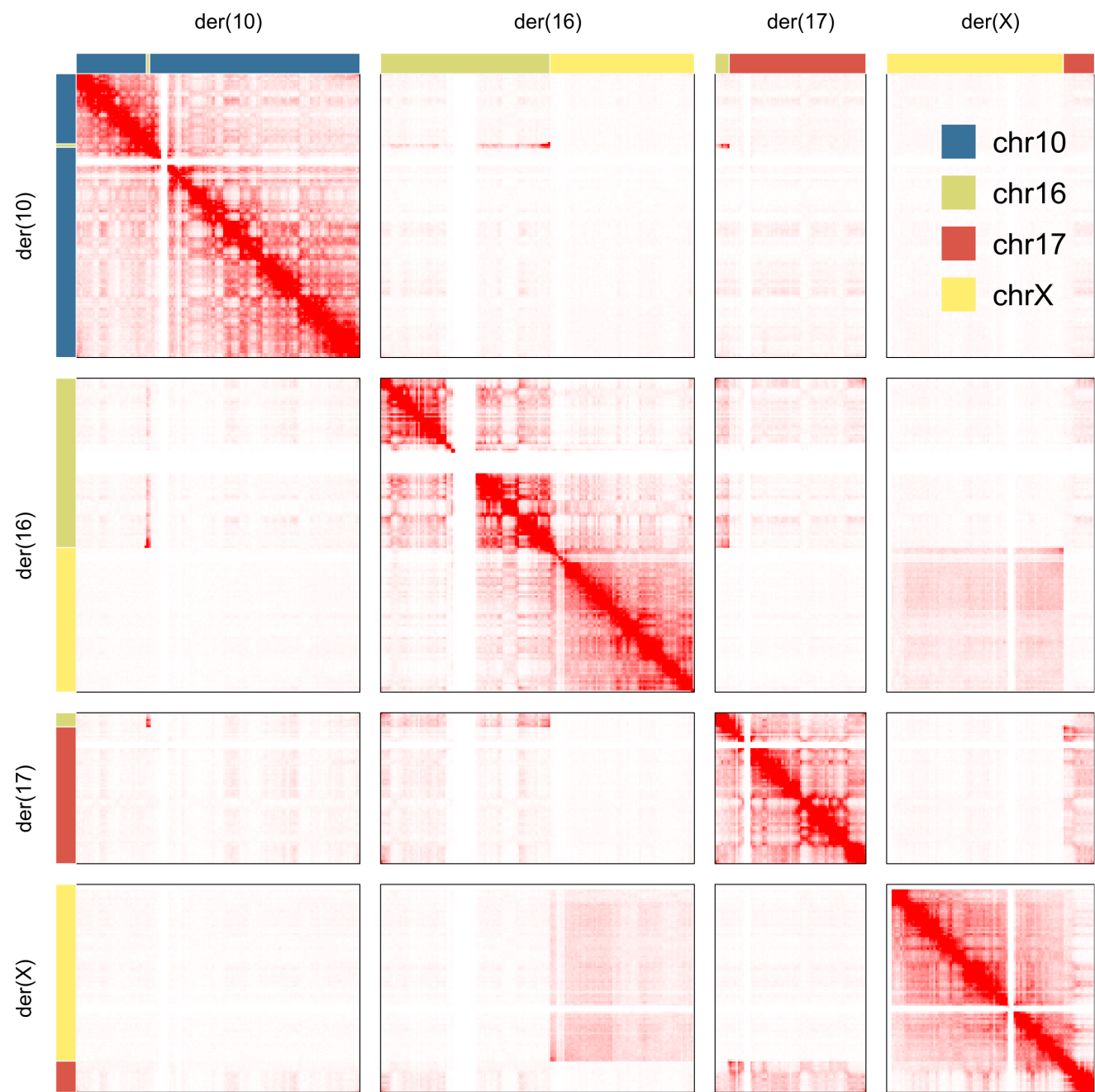

CP3

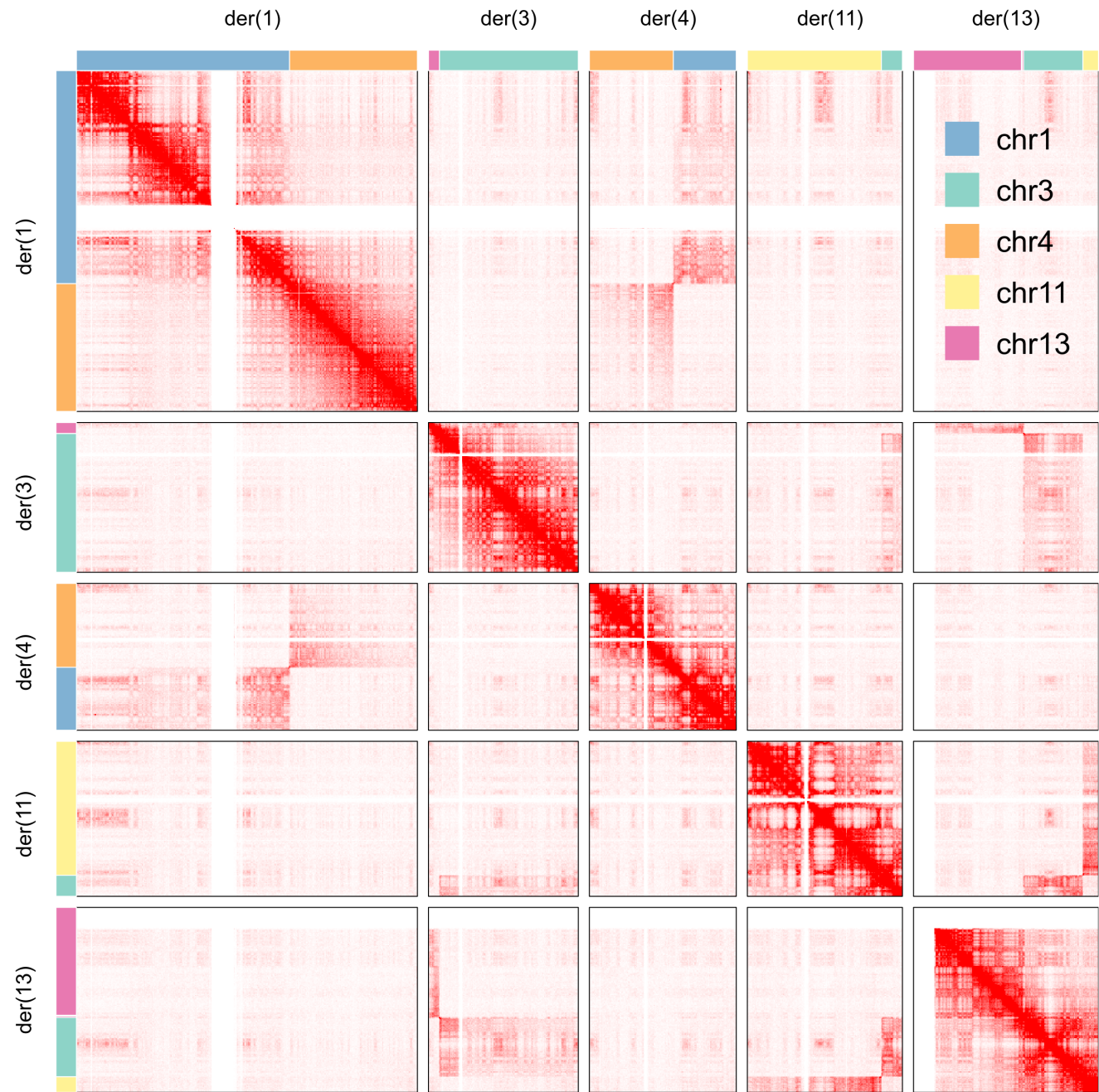

# CP4

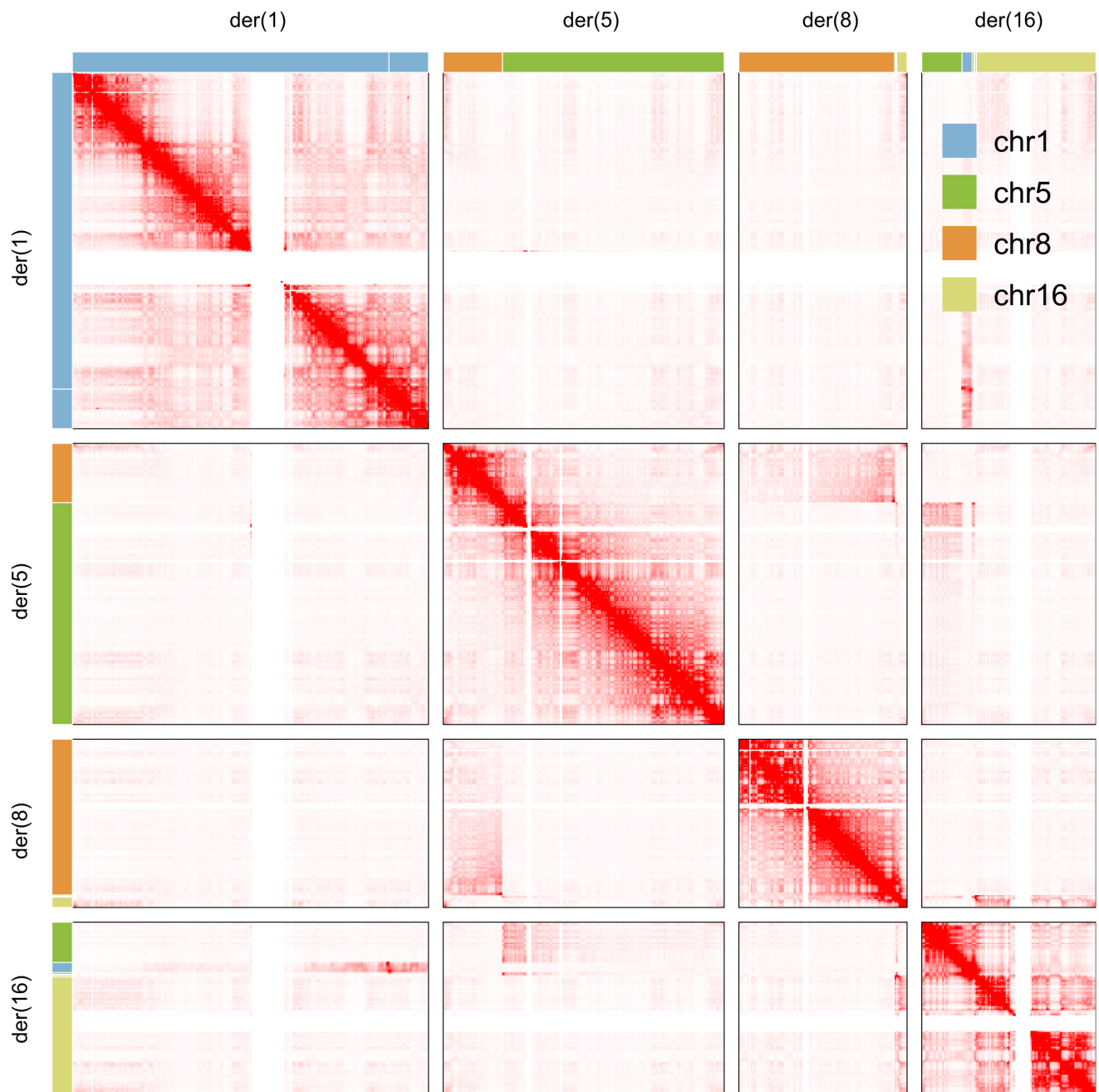

# CT1

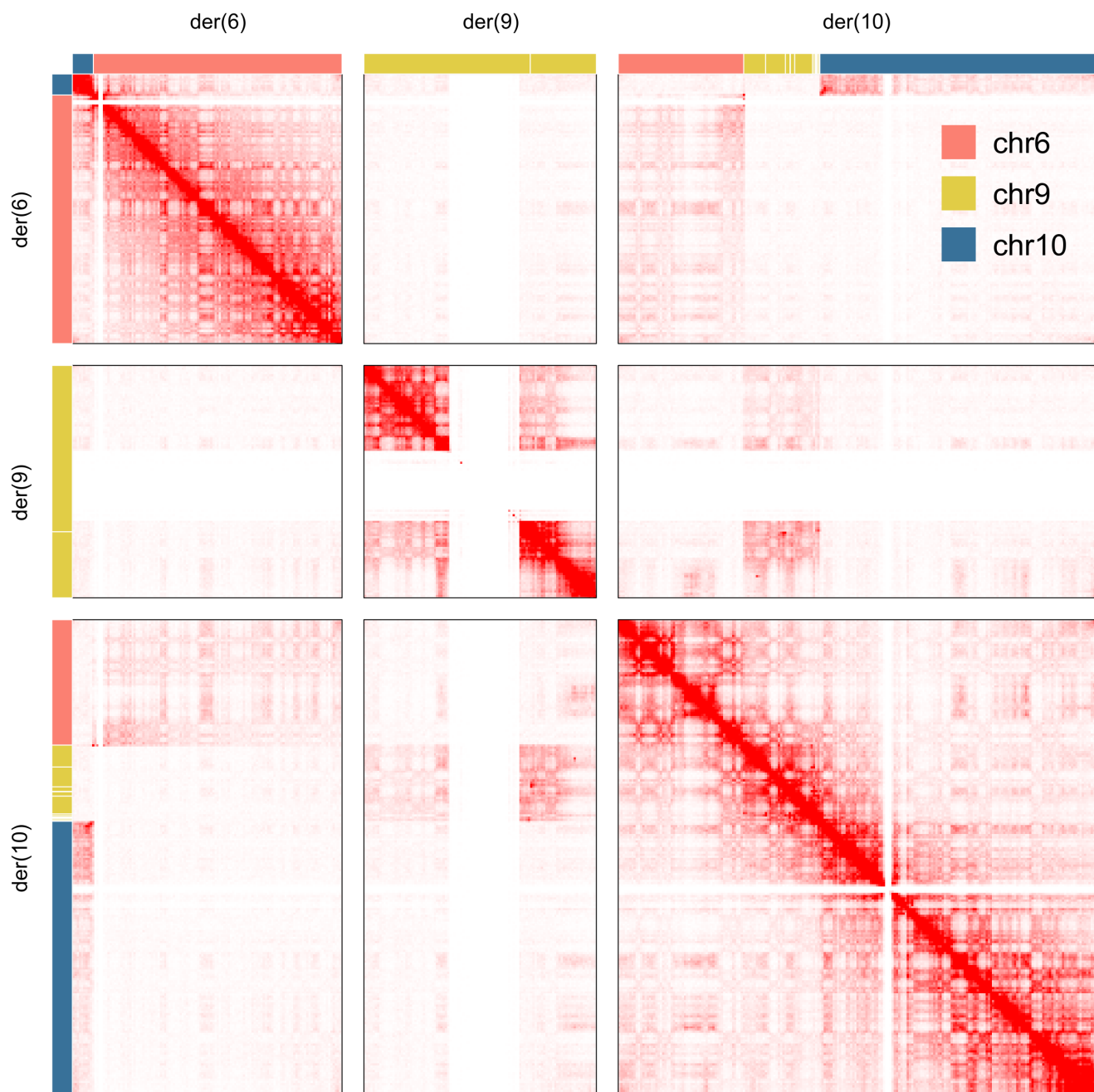

CT2

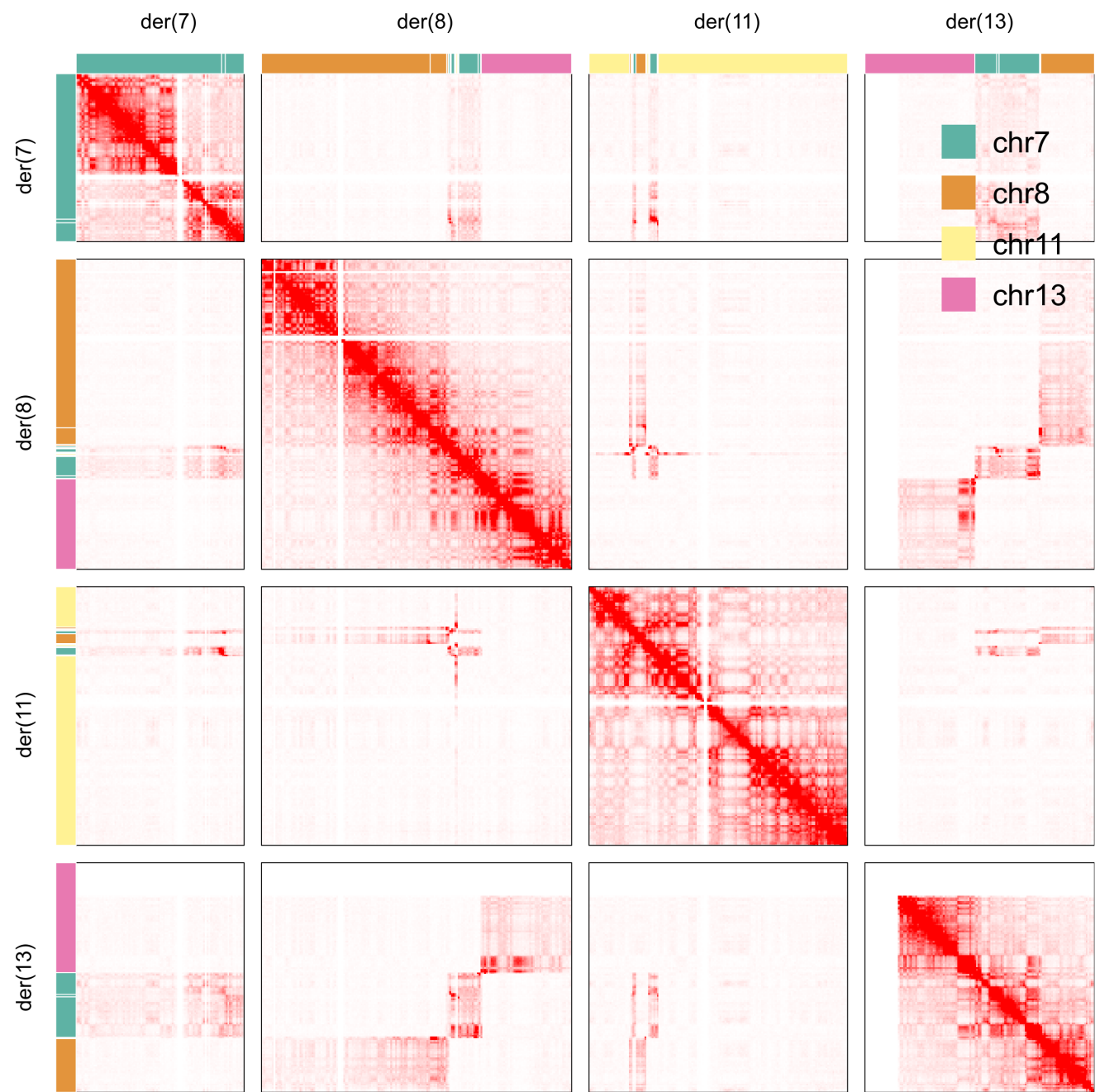

CT3

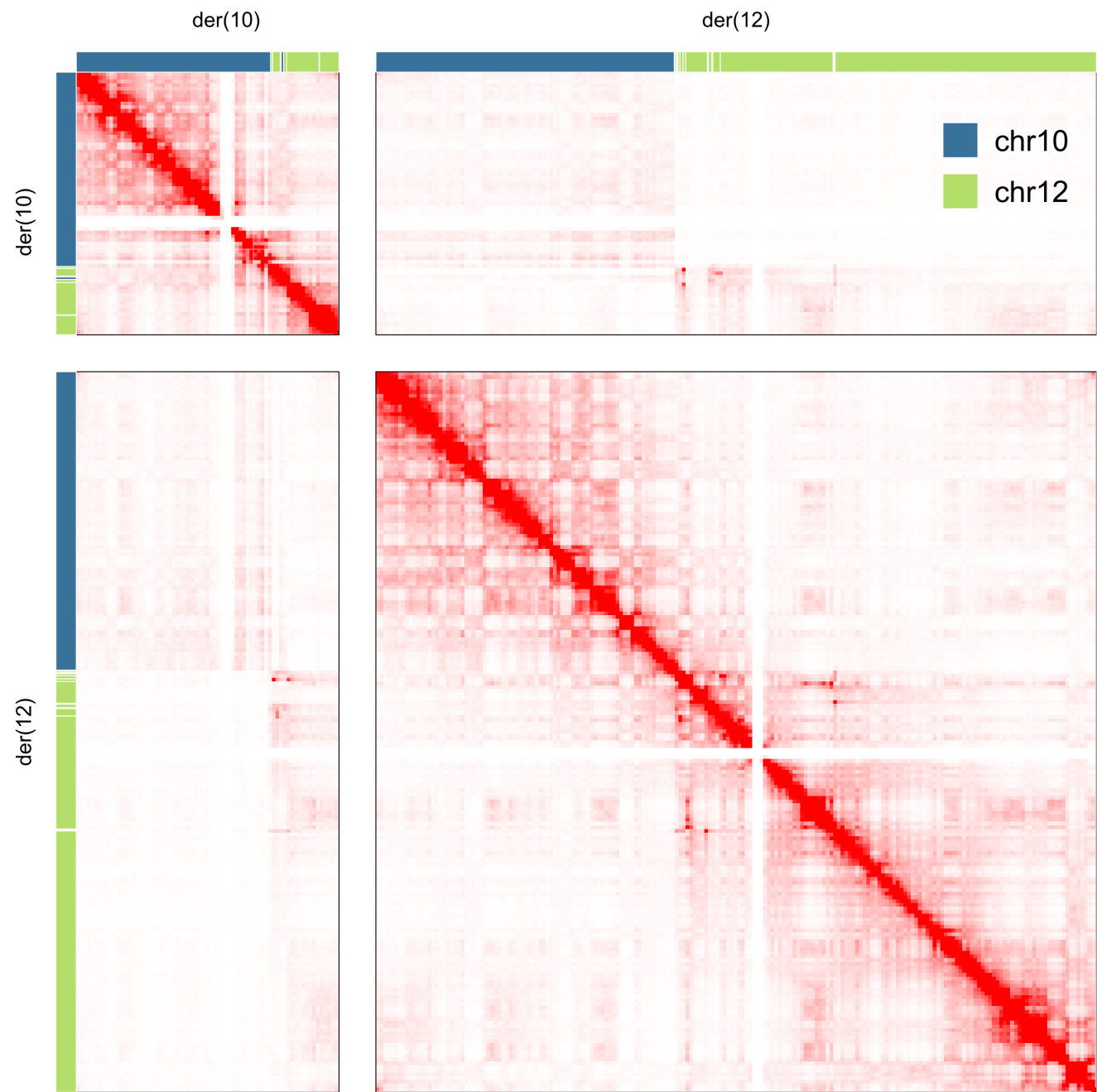

CT4

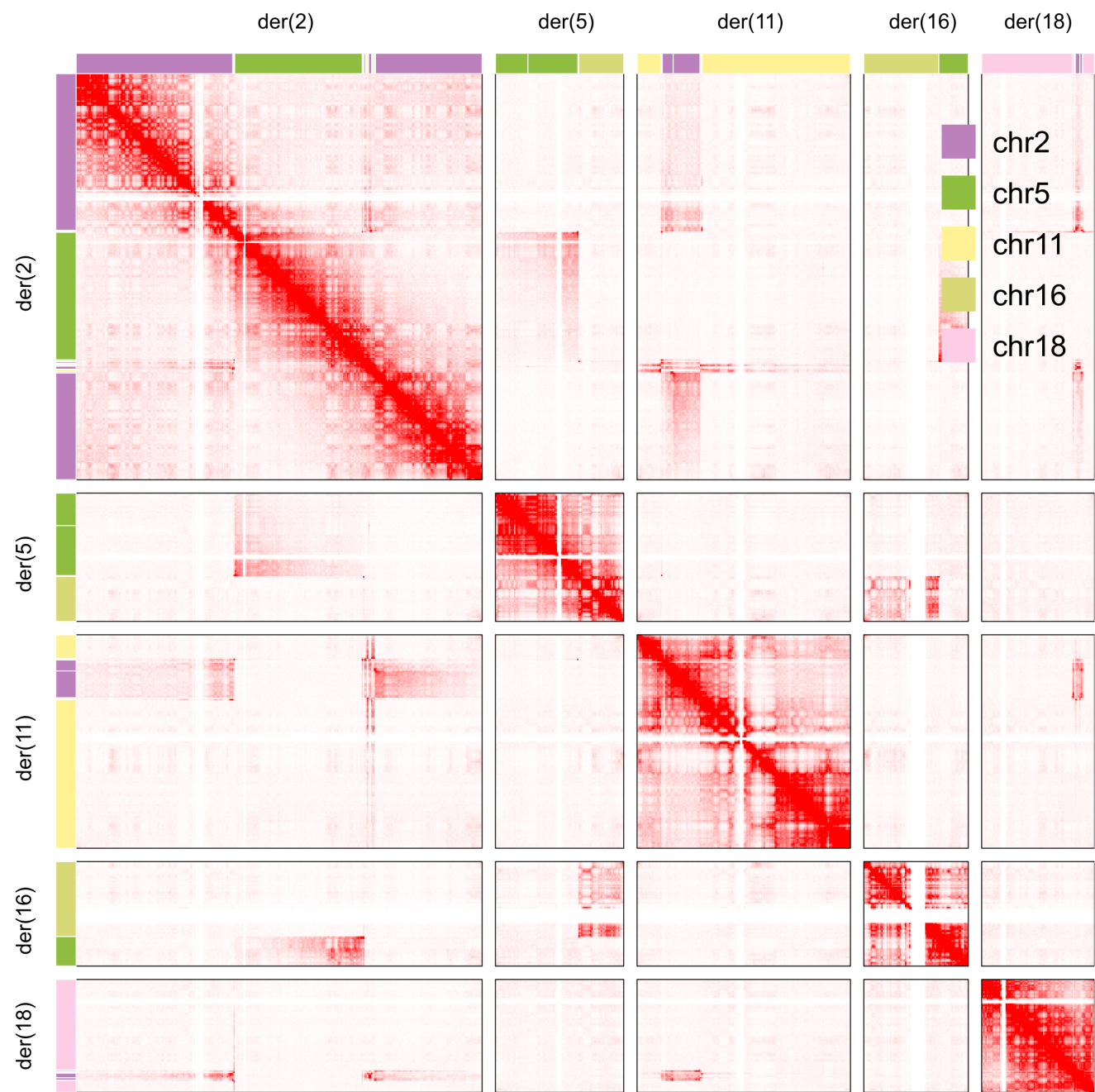

CT5

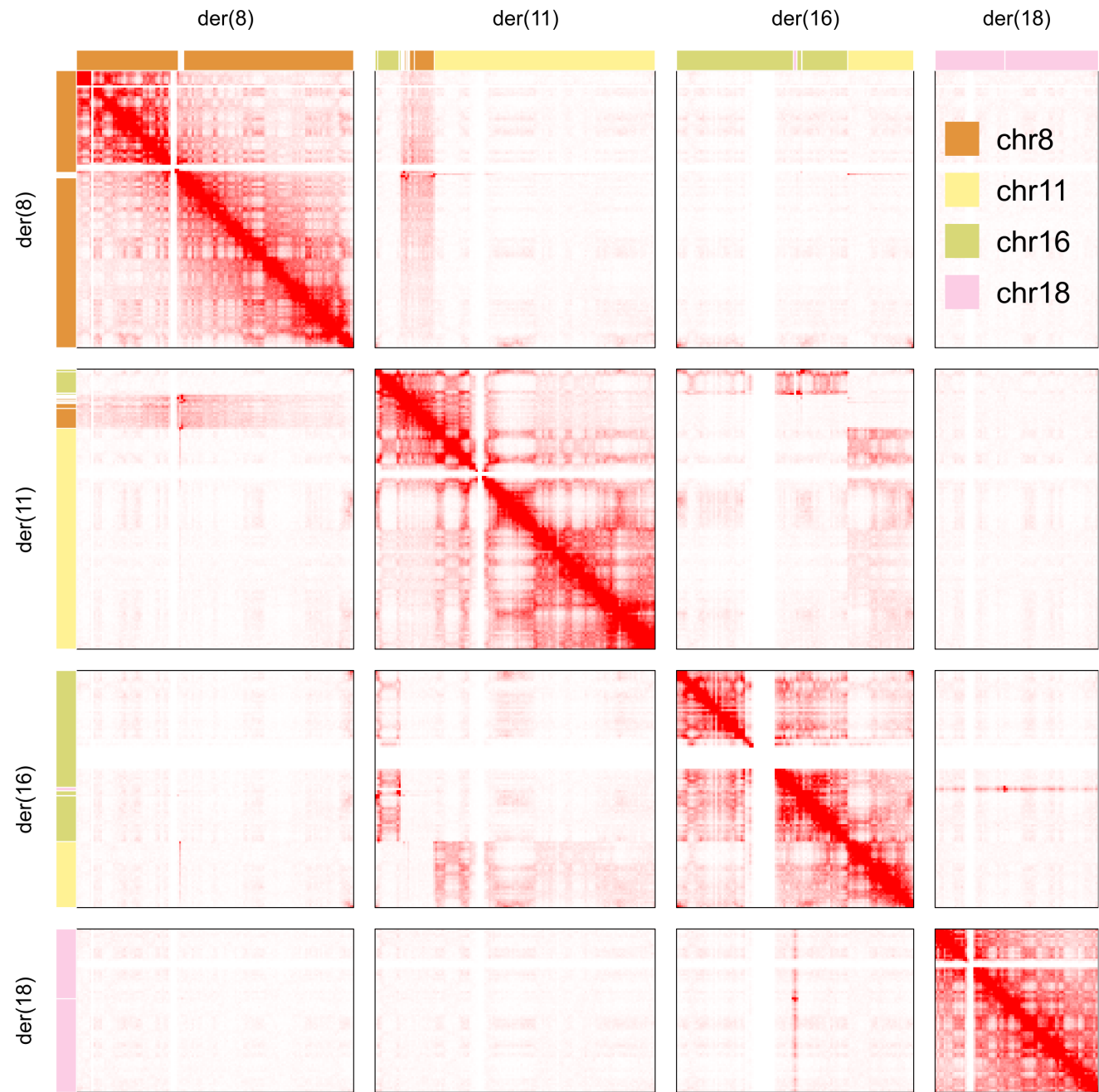

# CT6

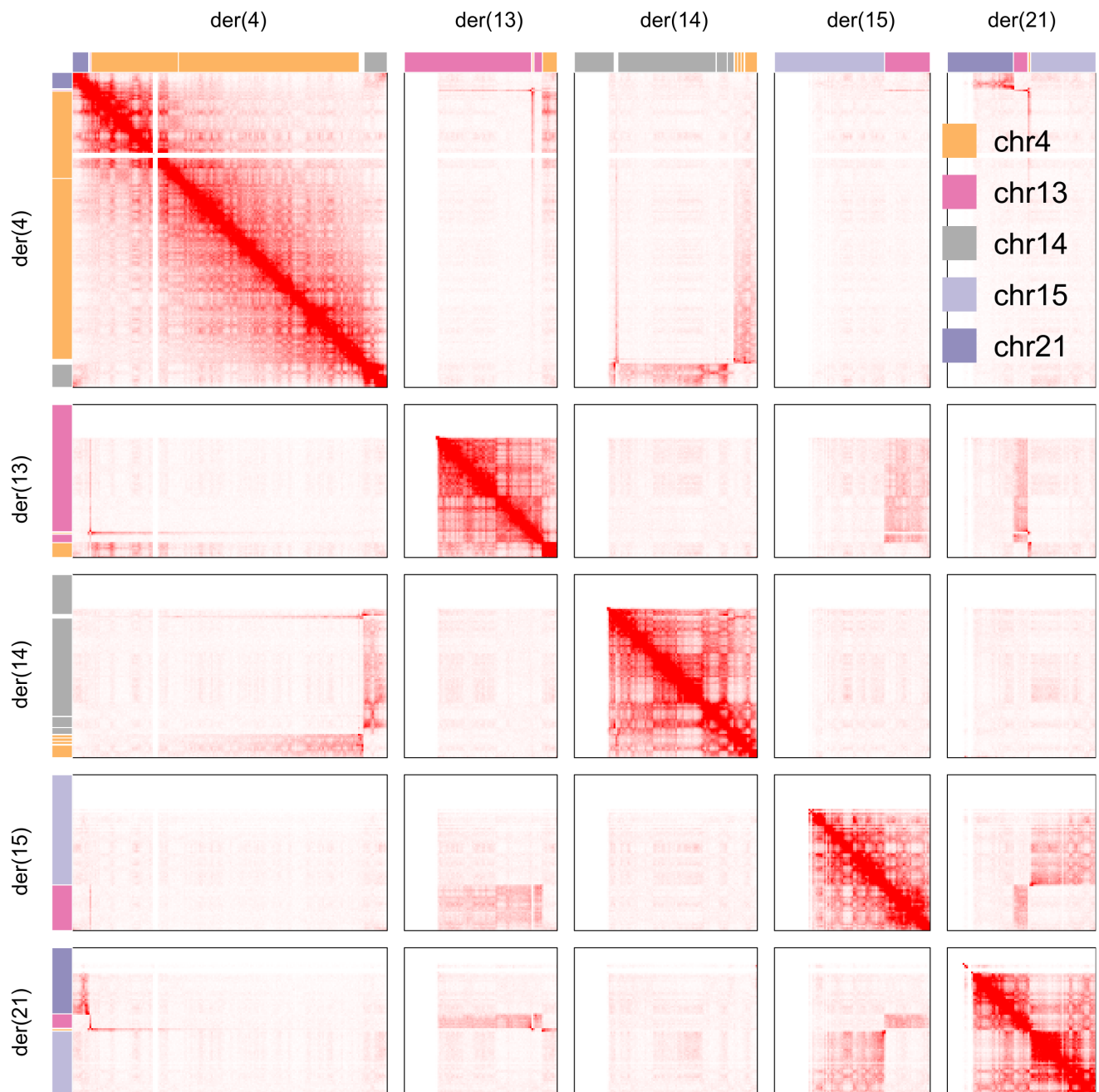

# CT7

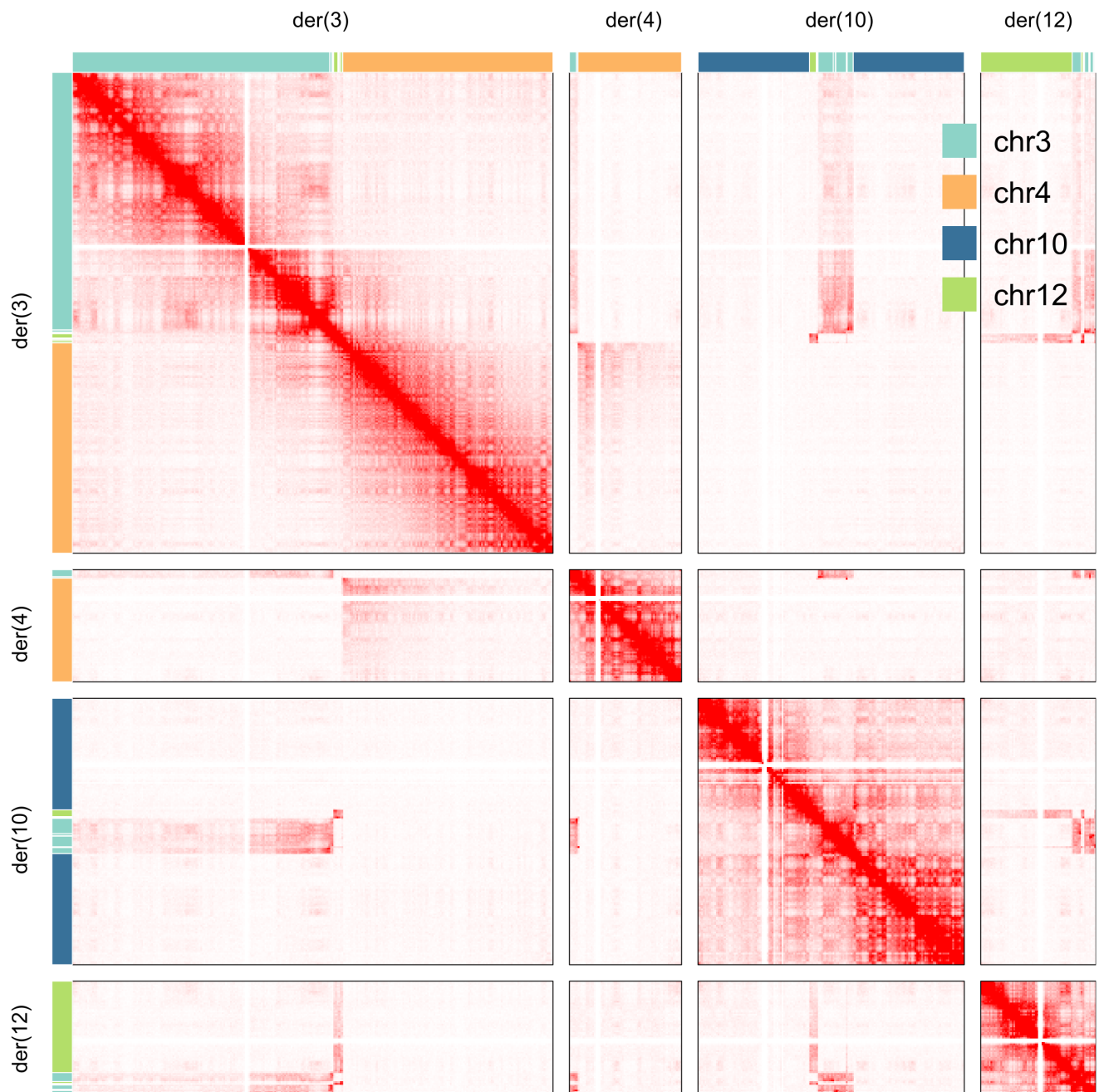

Supplement: Supplementary file 3 — Supplementary Data 2 [file 41467_2022_34053_MOESM3_ESM.pdf]
