# Supplementary material for: Integration of Hi-C with short and long-read genome sequencing reveals the structure of germline rearranged genomes
Source: Nat Commun. 2022 Oct 29;13:6470. doi: 10.1038/s41467-022-34053-7 (PMC9617858; doi:10.1038/s41467-022-34053-7)
Supplement: Supplementary file 4 — Reporting Summary [file 41467_2022_34053_MOESM4_ESM.pdf]

Corresponding author(s): Stefan Mundlos
Martin Vingron

Last updated by author(s): Sep 15, 2022

Reporting Summary

Nature Portfolio wishes to improve the reproducibility of the work that we publish. This form provides structure for consistency and transparency in reporting. For further information on Nature Portfolio policies, see our Editorial Policies and the Editorial Policy Checklist.

Statistics

For all statistical analyses, confirm that the following items are present in the figure legend, table legend, main text, or Methods section.

n/a	Confirmed	
[ ]	[x]	The exact sample size (*n*) for each experimental group/condition, given as a discrete number and unit of measurement
[ ]	[x]	A statement on whether measurements were taken from distinct samples or whether the same sample was measured repeatedly
[ ]	[x]	The statistical test(s) used AND whether they are one- or two-sided<br>*Only common tests should be described solely by name; describe more complex techniques in the Methods section.*
[x]	[ ]	A description of all covariates tested
[ ]	[x]	A description of any assumptions or corrections, such as tests of normality and adjustment for multiple comparisons
[ ]	[x]	A full description of the statistical parameters including central tendency (e.g. means) or other basic estimates (e.g. regression coefficient) AND variation (e.g. standard deviation) or associated estimates of uncertainty (e.g. confidence intervals)
[ ]	[x]	For null hypothesis testing, the test statistic (e.g. *F*, *t*, *r*) with confidence intervals, effect sizes, degrees of freedom and *P* value noted<br>*Give P values as exact values whenever suitable.*
[x]	[ ]	For Bayesian analysis, information on the choice of priors and Markov chain Monte Carlo settings
[x]	[ ]	For hierarchical and complex designs, identification of the appropriate level for tests and full reporting of outcomes
[x]	[ ]	Estimates of effect sizes (e.g. Cohen's *d*, Pearson's *r*), indicating how they were calculated

Our web collection on statistics for biologists contains articles on many of the points above.

Software and code

Policy information about availability of computer code

Data collection

No software was used.

Data analysis

* Mapping of Illumina short-reads from Hi-C and genome sequencing: bwa (version 0.7.17)
* Masking of duplicate reads from short-read genome sequencing: SAMBLASTER (version 0.1.24)
* Handling of sam-files from short-read genome sequencing: Samtools (version 1.9)
* Structural variation calling on short-read genome sequencing: Delly (version 0.8.1)
* Exporting data from BCF files to txt format: bcftools (version 1.10.2)

* Alignment of PacBio long-read genome sequencing data: pbmm2 (version 1.3.0)
* Structural variation calling on aligned long-read genome sequencing data: SVIM (version 1.4.1)
* Identification of gap locations in the reference sequence: seqtk (version 1.3)

* Processing of Hi-C data: Juicer pipeline (version 1.5.6, CPU version)
* Creation of Hi-C maps and data export: Juicer tools (version 1.7.5)
* Inspection of Hi-C maps and overlay with SV-calls: Juicebox (version 1.8.8, Desktop version)
* Custom code for the reconstruction of derivative chromosomes will be made available for the reviewers (https://github.molgen.mpg.de/schoepfl/chromosome_reconstruction)

* Alignment of RNA-seq data: STAR mapper (version 020201)
* Differential gene expression analysis: DESeq2 (version 1.26.0)
* Fusion transcript detection: Arriba (version 2.1.0)

* Whole genome haplotyping with PacBio long-reads and Hi-C short-reads: HapCUT2 (https://github.com/vibansal/HapCUT2)
* Calling of small variants for haplotyping: freebayes (version 1.2.0)
* Marking duplicates in Hi-C reads for haplotyping: Picard tools (version 2.20.8-0)
* Tagging of reads during haplotyping and phasing and converting HapCUT2 result to a vcf file: Whatshap (version 0.18)
* Custom code for haplotyping and phasing will be made available for the reviewers (https://github.com/moeinzadeh/Chromothripsis_haplotyping)

* Generation of Circos plots for rearranged chromosomes: R-package Circlize (version 0.4.13)
* Computation of short-read coverage from genome sequencing for chromosomal fragments: R-package bamsignals (version 1.18.0)
* Color palettes were obtained with the help of the online tools https://colorbrewer2.org and https://paletton.com

* Analysis of breakpoint signature: Python-package pysam (version 0.15.2) and Python-package biophython (version 1.73)
* Visualization of mapped long reads: IGV 2.9.2
* Visualization of examples of genes and breakpoints: UCSC genome browser (Website)

* Analysis of genomic intervals: R-package GenomicRanges (version 1.38.0)
* Creation of plots: R (mainly) , and python

For manuscripts utilizing custom algorithms or software that are central to the research but not yet described in published literature, software must be made available to editors and reviewers. We strongly encourage code deposition in a community repository (e.g. GitHub). See the Nature Portfolio guidelines for submitting code & software for further information.

Data

Policy information about availability of data

All manuscripts must include a data availability statement. This statement should provide the following information, where applicable:

- Accession codes, unique identifiers, or web links for publicly available datasets
- A description of any restrictions on data availability
- For clinical datasets or third party data, please ensure that the statement adheres to our policy

The informed consents do not cover the deposition of sequencing data from the patient samples.

Field-specific reporting

Please select the one below that is the best fit for your research. If you are not sure, read the appropriate sections before making your selection.

- [x] Life sciences
- [ ] Behavioural & social sciences
- [ ] Ecological, evolutionary & environmental sciences

For a reference copy of the document with all sections, see nature.com/documents/nr-reporting-summary-flat.pdf

Life sciences study design

All studies must disclose on these points even when the disclosure is negative.

Sample size	No sample size calculation was performed. Statistical testing between groups was not performed in this study. The cases of complex genomic rearrangements investigated here are very rare and it is difficult to recruit probands. This set the limit for the sample size.
Data exclusions	The sample of case CT2 was not included in downstream analysis of allelic imbalance genes, because of the high base level of genes identified as allelic imbalanced in this sample.
Replication	Hi-C libraries were prepared as 2-4 technical replicates. All replicates were used for the analysis. The replicates of each case were merged after the Hi-C data processing. RNA-seq was performed in three biological replicates, which were jointly analyzed in the differential gene expression analysis. All replicates were used for the analysis. For case CT5, only 2 replicates were available.
Randomization	No randomization was applied in this study. Statistical testing between groups was not performed in this study.
Blinding	No blinding was applied in this study. Statistical testing between groups was not performed in this study.

Reporting for specific materials, systems and methods

We require information from authors about some types of materials, experimental systems and methods used in many studies. Here, indicate whether each material, system or method listed is relevant to your study. If you are not sure if a list item applies to your research, read the appropriate section before selecting a response.

Materials & experimental systems

n/a	Involved in the study
☒	☐ Antibodies
☐	☒ Eukaryotic cell lines
☒	☐ Palaeontology and archaeology
☒	☐ Animals and other organisms
☐	☒ Human research participants
☒	☐ Clinical data
☒	☐ Dual use research of concern

Methods

n/a	Involved in the study
☒	☐ ChIP-seq
☒	☐ Flow cytometry
☒	☐ MRI-based neuroimaging

Eukaryotic cell lines

Policy information about cell lines

Cell line source(s)	Peripheral blood lymphocytes were used for establishing lymphoblastoid cell lines by EBV transformation for 10 cases. For one case, a fibroblast cell line was established.
Authentication	All cell lines used here were derived from the individuals` enrolled for this study.
Mycoplasma contamination	Cell lines were not tested for mycoplasma contamination.
Commonly misidentified lines (See ICLAC register)	Not applicable, all cell lines used here were derived from the individuals` enrolled for this study.

Human research participants

Policy information about studies involving human research participants

Population characteristics	The human research participants were recruited based the occurrence of large-scale chromosomal rearrangements. Samples from males and females with different phenotypes and varying age were included. Gene expression differences were compared between the alleles of the same individual, but not between individuals.
Recruitment	From our in-house cohort of individuals with chromosomal rearrangements detected by karyotyping, we selected seven cases with > 3 chromosomal rearrangements to be enrolled in this study. Through collaborative efforts, we obtained additional four cases.
Ethics oversight	Informed consent to publish genomic and clinical data was obtained from all patients (or their legal guardian). The study protocol was approved by the local ethics committee "Ethikkommission der Charité", Berlin, Germany, and followed relevant ethical regulations.

Note that full information on the approval of the study protocol must also be provided in the manuscript.
